# Direct, gabapentin-insensitive interaction of a soluble form of the calcium channel subunit α_2_δ-1 with thrombospondin-4

**DOI:** 10.1038/s41598-019-52655-y

**Published:** 2019-11-07

**Authors:** Ehab El-Awaad, Galyna Pryymachuk, Cora Fried, Jan Matthes, Jörg Isensee, Tim Hucho, Wolfram F. Neiss, Mats Paulsson, Stefan Herzig, Frank Zaucke, Markus Pietsch

**Affiliations:** 1 Institute II for Pharmacology, Centre of Pharmacology, Medical Faculty, University of Cologne, Gleueler Str. 24, D-50931 Cologne, Germany; 2 Department of Pharmacology, Faculty of Medicine, Assiut University, Assiut, 71515 Egypt; 3 Department of Anatomy I, Medical Faculty, University of Cologne, Kerpener Str. 62, D-50937 Cologne, Germany; 4 Experimental Anaesthesiology and Pain Research, Department of Anaesthesiology and Intensive Care Medicine, Medical Faculty, University of Cologne, Robert-Koch-Str. 10, D-50931 Cologne, Germany; 5 Institute for Biochemistry II, Centre for Biochemistry, Medical Faculty, University of Cologne, Joseph-Stelzmann-Str. 52, D-50931 Cologne, Germany; 6 Centre for Molecular Medicine Cologne (CMMC), University of Cologne, Robert-Koch-Str. 21, D-50931 Cologne, Germany; 7 President of TH Köln, TH Köln (University of Applied Sciences), Claudiusstr. 1, D-50678 Cologne, Germany; 8 Dr. Rolf M. Schwiete Research Unit for Osteoarthritis, Orthopedic University Hospital, Friedrichsheim gGmbH, Marienburgstr. 2, D-60528 Frankfurt/Main, Germany

**Keywords:** Membrane proteins, Biochemical assays, Protein purification, Mass spectrometry, Chronic pain

## Abstract

The α_2_δ‐1 subunit of voltage-gated calcium channels binds to gabapentin and pregabalin, mediating the analgesic action of these drugs against neuropathic pain. Extracellular matrix proteins from the thrombospondin (TSP) family have been identified as ligands of α_2_δ‐1 in the CNS. This interaction was found to be crucial for excitatory synaptogenesis and neuronal sensitisation which in turn can be inhibited by gabapentin, suggesting a potential role in the pathogenesis of neuropathic pain. Here, we provide information on the biochemical properties of the direct TSP/α_2_δ-1 interaction using an ELISA-style ligand binding assay. Our data reveal that full-length pentameric TSP-4, but neither TSP-5/COMP of the pentamer-forming subgroup B nor TSP-2 of the trimer-forming subgroup A directly interact with a soluble variant of α_2_δ-1 (α_2_δ-1_S_). Interestingly, this interaction is not inhibited by gabapentin on a molecular level and is not detectable on the surface of HEK293-EBNA cells over-expressing α_2_δ‐1 protein. These results provide biochemical evidence that supports a specific role of TSP-4 among the TSPs in mediating the binding to neuronal α_2_δ‐1 and suggest that gabapentin does not directly target TSP/α_2_δ-1 interaction to alleviate neuropathic pain.

## Introduction

**Figure 1:**
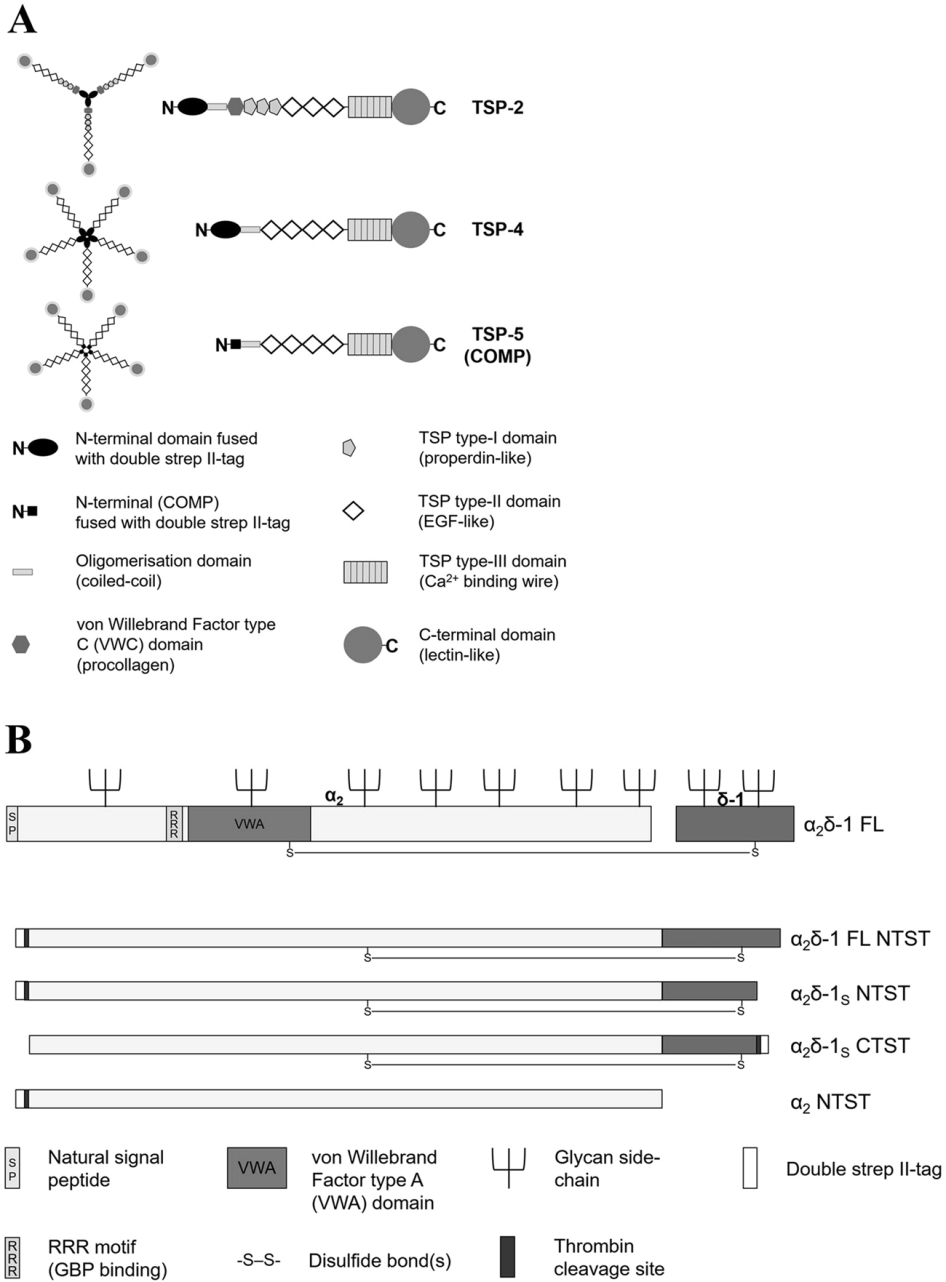
Schematic presentation of the structures of the recombinant proteins generated in this study. (**A**) Domain structure and oligomerisation state of the generated recombinant full-length TSP-2 (trimer), TSP-4 and COMP (pentamers). Schematic representation adapted by permission from Springer Nature, *Cell Mol Life Sci*, Structures of thrombospondins, Carlson, C. B., Lawler, J. & Mosher, D. F., Copyright (2008)^39^. All recombinant TSPs have been expressed with an N-terminal double strep II-tag and contain glycan side-chains which are not shown for reasons of clarity. (**B**) Structure of α_2_δ-1 FL protein (adapted from *Cell* **139**, Eroglu, Ç. *et al*., Gabapentin receptor αδ2δ-1 is a neuronal thrombospondin receptor responsible for excitatory CNS synaptogenesis, 380–392, Copyright (2009), with permission from Elsevier^7^) and simplified depiction of the derived non-proteolytically processed α_2_δ-1 mutants generated in this study. The RRR motif, the von Willebrand Factor type A domain, and the glycan side-chains are not shown in the α_2_δ-1 mutants for reasons of clarity.

Thrombospondins (TSPs) form a family of five large oligomeric extracellular matrix glycoproteins that are expressed by numerous cell types, playing important roles in cellular migration, attachment and cytoskeletal dynamics^1,2^. Several TSP isoforms have been shown to be involved in a variety of physiological and pathological processes, including regulation of angiogenesis, apoptosis and platelet aggregation^3–5^. TSPs can be subdivided into subgroups A (TSP-1 and 2) and B (TSP-3–5, with TSP-5 also referred to as cartilage oligomeric matrix protein (COMP)) based on their oligomerisation state (trimeric or pentameric, respectively) and domain structure (Fig. 1A). In neurons, astrocyte-secreted or recombinantly expressed TSP(s), particularly TSP-1, TSP-2 and TSP-4, were reported to promote the formation of excitatory synapses both *in vitro* and *in vivo* through interaction with the voltage-gated calcium channel subunit α_2_δ-1^6–10^. The α_2_δ proteins (α_2_δ‐1–4) are auxiliary subunits of voltage-gated calcium channels Ca_*V*_1 and Ca_*V*_2, and were found to be encoded by four different genes^11–13^. Functions of these auxiliary subunits include the modulation of trafficking, expression in the plasma membrane^14–17^, and biophysical properties of the channels^15,17–19^. Importantly, α_2_δ-1 acts as a specific binding site for gabapentinoid drugs^20,21^, mediating their analgesic effect in neuropathic pain^21–23^. Furthermore, studies using different animal models of neuropathic pain indicated the involvement of α_2_δ-1 in pain development, with nerve injuries leading to up-regulation of α_2_δ‐1 in both dorsal root ganglion (DRGs) and spinal dorsal horn neurons^24–27^ as well as to an increase of miniature excitatory post-synaptic current (mEPSC) frequency in the latter neurons^22,25,26,28–30^. Similarly, injury-induced TSP-4 is reported to mediate central sensitisation and neuropathic pain states^8,9,31–37^. This effect was recently shown to be mediated by activation of a TSP-4/α_2_δ‐1-dependent pathway which requires a direct molecular interaction between the two proteins^9,34^. Furthermore, the presence of TSP-4 was shown to modestly but significantly reduce the binding affinity of ^3^H-gabapentin (^3^H-GBP) towards α_2_δ‐1 in membrane preparations from TSP-4/α_2_δ‐1 co-transfected cells^38^. Taken together, the TSP-4/α_2_δ‐1 protein-protein interaction seems to be of potential translational importance and thus may serve as a novel target for developing a new class of analgesics against neuropathic pain.

The aim of the present study is to investigate the biochemical characteristics of the direct molecular interaction between TSPs and α_2_δ‐1, addressing the question whether α_2_δ‐1 binding is specific to TSP-4 or redundant among other TSPs. GBP has been shown so far to inhibit the interaction of α_2_δ‐1 with a truncated form of TSP-2 in co-immunoprecipitation experiments^7^ as well as functionally by inhibiting synaptogenesis^7,8,10^, neuron sensitisation and behavioural hypersensitivity induced by TSP-2, its truncated fragment and/or TSP-4^9,34,35^. Thus, we examined whether the direct TSP/α_2_δ‐1 interaction can be inhibited by GBP on a molecular level as well. We therefore generated purified recombinant forms of three full-length TSPs (TSP-2, TSP-4 and COMP) as well as soluble forms of α_2_δ‐1 subunit (α_2_δ‐1_S_), that shows GBP binding affinity similar to that of wild-type α_2_δ-1, and the α_2_ peptide chain of α_2_δ‐1 (Fig. 1). Both the interaction of these recombinant TSPs with α_2_δ‐1 and the possible inhibition by GBP were examined in a solid-phase ELISA-style ligand binding assay, with the capability of soluble α_2_δ‐1 to interact with GBP being proven by a newly developed surface plasmon resonance (SPR)-based binding assay. In order to demonstrate the characteristics of the direct TSP-4/α_2_δ‐1 interaction in an environment similar to that of native cells, we attempted to visualise the interaction of fluorescently labelled TSP-4 with membrane-localised full-length (FL) α_2_δ‐1 in a cell-based system.

## Results

### Biochemical characteristics of recombinant purified proteins expressed in HEK293-EBNA cells

**Figure 2:**
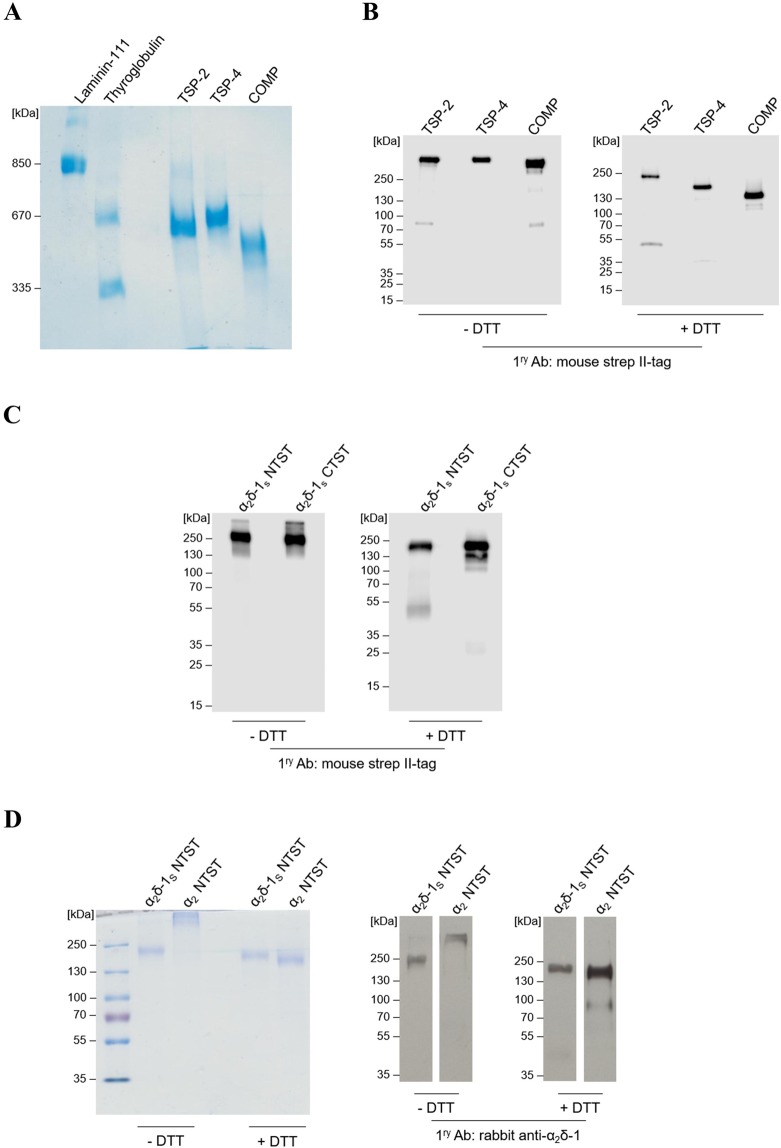
The generated recombinant TSPs and α_2_δ-1_S_ variants show high degree of purity and integrity in Coomassie staining and western blot analyses. (**A**,**D** left) Representative Coomassie-stained gels and (**B,C** and **D** right) immunoblots of three full-length TSP proteins, all carrying an N-terminal double strep II-tag: TSP-2, TSP-4, and COMP (**A,B**); α_2_δ-1_S_ variants carrying either an N-terminal (α_2_δ-1_S_ NTST) or a C-terminal (α_2_δ-1_S_ CTST) double strep II-tag (**C**); α_2_δ-1_S_ NTST and α_2_ peptide chain carrying an N-terminal double strep II-tag, α_2_ NTST (**D**). Proteins were separated under non-reducing (−DTT) or reducing conditions (+DTT) on 4–15% (**B**), 10% (**C**), and 7% (**D**) polyacrylamide gels, respectively, while in (**A**) proteins were separated on 0.5% agarose (w/v)/3% polyacrylamide (w/v) composite gels without prior DTT treatment. Proteins were either stained with colloidal Coomassie stain (**A,D** left) or detected with the following primary antibodies after blotting: mouse anti-strep II-tag (**B,C**) or rabbit anti-α_2_δ-1 (**D** right). Secondary antibodies included the polyclonal rabbit anti-mouse IgG (**B,C**) and swine anti-rabbit IgG (**D** right), both conjugated with horseradish peroxidase (see Supplementary Table S2 for further information). In all gels the molecular weight standard (in kDa) indicated on the left was PageRuler Plus Prestained Protein Ladder (Thermo Fisher Scientific) except for (**A**) in which both thyroglobulin (Sigma) and recombinant laminin-111 (kind gift from Prof. Dr. Monique Aumailley, Institute for Biochemistry II, Centre for Biochemistry, Medical Faculty, University of Cologne) were used.

To investigate the direct binding of different TSPs to α_2_δ-1, three full-length recombinant TSPs, namely, the trimeric TSP-2, the pentameric proteins TSP-4 and COMP, and a soluble C-terminal deletion mutant of α_2_δ-1 carrying an N-terminal (α_2_δ-1_S_ NTST) or a C-terminal (α_2_δ-1_S_ CTST) double strep II-tag (Fig. 1) were generated in a eukaryotic expression system. Coomassie stained gels and immunoblots of the purified proteins confirmed their purity, identity and integrity (Fig. 2; Supplementary Fig. S1). As expected for TSPs, the intact proteins showed high molecular weight bands with approximate apparent molecular weights (*M*r) in the range ~500–670 kDa when compared to laminin-111 and thyroglobulin as marker proteins (Fig. 2A). The oligomerisation patterns of these high molecular weight TSPs (*i.e*. pentamers for TSP-4 and COMP and trimer for TSP-2) were confirmed by comparing immunoblots in the absence and presence of the reducing reagent DTT (Fig. 2B; Supplementary Fig. S1a). In the latter case, DTT reduces the interchain disulphide bonds within the oligomerisation domains of the analysed TSPs^39^ and major bands of monomeric proteins with *M*r of TSP-2 (~240 kDa), TSP-4 (~160 kDa), and COMP (~130 kDa) were observed (Fig. 2B, +**DTT**; Supplementary Fig. S1a, **+DTT**).

Similar analysis was performed for the generated α_2_δ-1_S_ NTST and the respective C-terminally tagged α_2_δ-1 variant, α_2_δ-1_S_ CTST, showing single but smeared bands at approximate *M*r ~200 kDa under non-reducing conditions (Fig. 2C, **−DTT**; Supplementary Fig. S1b, −**DTT**) and appear as distinct bands at approximate *M*r ~180 kDa under reducing conditions (Fig. 2C, **+DTT**; Supplementary Fig. S1b, **+DTT**). So far, there is no comprehensive explanation for this gel band shift in the presence of DTT. However, it cannot be attributed to the reductive cleavage of the interchain disulphide bridge between α_2_ and δ-1 followed by loss of the smaller δ-1 chain. This conclusion arises from the observation that α_2_δ-1_S_ bearing the C-terminal double strep II-tag (α_2_δ-1_S_ CTST) is still detectable in immunoblots probed with strep II-tag antibody following DTT treatment. In agreement with this result, mass spectra of α_2_δ-1_S_ NTST recorded with and without DTT pre-treatment showed almost identical molecular ion peaks ([M + H]^+^; Table 1, Supplementary Fig. S2d,e). This observation confirms the results by Brown and Gee^40^ who first described a similar soluble mutant of the porcine α_2_δ-1 orthologue which retains high affinity for ^3^H-GBP. Although uncleaved α_2_δ-1 may not represent a functional form as a subunit of the Ca_*V*_ channels and can inhibit native calcium currents in mammalian neurons^41^, the TSP/α_2_δ-1 pathway is thought to be at least partially independent of the roles of α_2_δ-1 as a Ca_V_ channel subunit^7,10^. Therefore, the recombinant uncleaved α_2_δ-1_S_ variant used in this study should be suitable for the purpose of investigating TSP binding biochemically. Notably, we observed a minor band in the immunoblots of α_2_δ-1_S_ CTST at *M*r ~25 kDa upon DTT treatment and detection with strep II-tag antibody (Fig. 2C, **+DTT**) which is most likely attributed to cleaved strep-tagged δ-1_S_ chain. This indicates the presence of a small fraction of the generated purified α_2_δ-1_S_ in a proteolytically cleaved form.

**Table 1:** Molecular masses of recombinant TSPs and α_2_δ-1_S_ NTST obtained by MALDI-TOF MS.

Protein	*m/z*_calc_ [M + H]^+^	*m/z*_exp_ [M + H]^+^
TSP-2 (monomer)	132,137	146,958
TSP-4 (monomer)	107,409	109,351
COMP (monomer)	84,714	88,463; 87,178
α_2_δ-1_S_ NTST (−DTT)	123,000	152,236
α_2_δ-1_S_ NTST (+DTT)	112,130^*^	152,258

The *m/z*_calc_ [M + H]^+^ values for all proteins (in Da) were calculated based on their amino acid sequences using ExPASy Compute pI/Mw online tool, while the *m/z*_exp_ [M + H]^+^ values (in Da) were obtained from the MALDI-TOF MS spectra of the respective proteins.

^*^ *m/z*_calc_ [M + H]^+^ of α_2_ NTST was calculated for the expected product of the DTT-mediated reduction of the disulphide bond between α_2_ and δ-1_S_ in α_2_δ-1_S_ NTST.

In addition to the α_2_δ-1_S_ variants generated, the α_2_ peptide chain (α_2_ NTST) was recombinantly produced in a similar way (Fig. 1B). Expression and purification of this fragment as well as analyses by SDS-PAGE and immunoblotting (Fig. 2D) were carried out as described above. Here, we observed the formation of a high molecular weight product under non-reducing conditions which dissociated into the monomeric form after DTT treatment (approximate *M*r ~ 170 kDa, Fig. 2D) which points to the formation of interchain disulphide bonds in the absence of reducing agents (see also Discussion section below).

Notably, all recombinant proteins generated in this study that had been analysed by SDS-PAGE and Western Blot showed protein bands at remarkably higher *M*r than expected from their amino acid sequences. It is known that the electrophoretic mobility of proteins can be greatly influenced by the extent of post-translational modifications (e.g. glycosylation) of the protein where the glycan chains do not bind SDS leading in many cases to decreased mobility, and increased *M*r, of the glycoprotein analysed by SDS-PAGE^42^. In addition, sample treatment prior to loading onto the gel (e.g. heating at 95 °C with DTT) represents a possible source of abnormal protein migration on the gels through its impact on the structure of the analysed protein^43^. Therefore, further analysis was carried out to determine the accurate molecular masses of the recombinant purified proteins using MALDI-TOF mass spectrometry. The results are shown in Table 1 and Supplementary Fig. S2. The molecular masses of TSP-4 and COMP, both in monomeric form, were found to be only slightly higher (1–3 kDa) than the theoretical masses calculated on the basis of each protein’s amino acid sequence, indicating minor post-translational modifications, e.g. glycosylation, of these proteins. In case of TSP-2 and α_2_δ-1_S_ NTST, the experimentally determined masses were about 15 and 30 kDa, respectively, larger than the theoretical ones, which is likely attributed to heavy glycosylation, as shown previously for α_2_δ-1 in the work of Kadurin *et al*.^44^ (Table 1, Supplementary Fig. S2a,d,e).

### Characterisation of the interaction of TSPs with α_2_δ-1_S_ using an ELISA-style ligand binding assay

**Figure 3:**
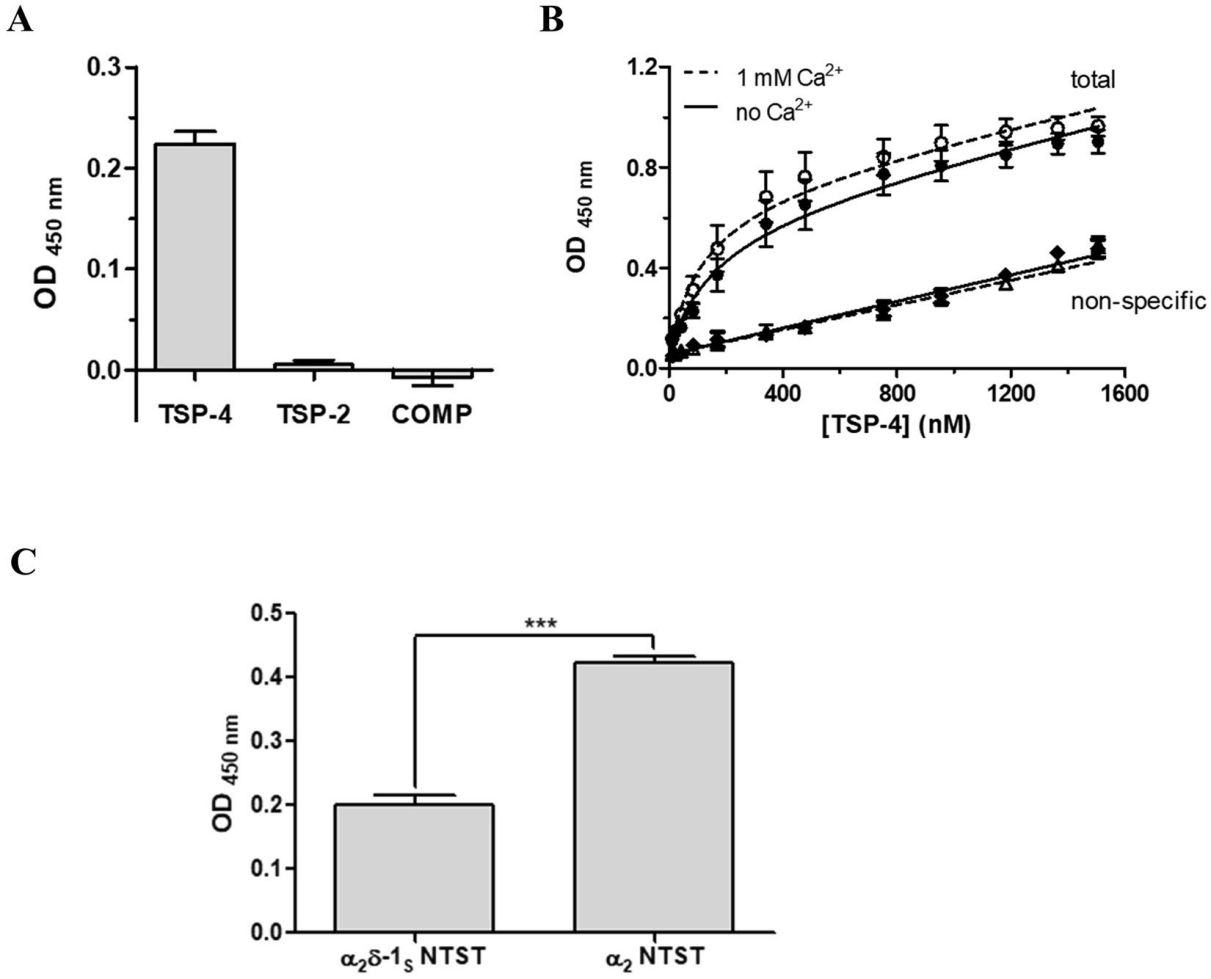
The direct binding to α_2_δ-1_S_ NTST is TSP-4 specific in an ELISA-style ligand binding assay. α_2_δ-1_S_ NTST (20 µg/ml) was coated onto 96-well plates and incubated with either (**A**) TSP-2, TSP-4 or COMP (1,000 nM), or (**B**) increasing concentrations of TSP-4 (11–1,505 nM). Shown are data for total (circles) and non-specific (triangles, rhombi) binding in the absence (full symbols) and presence (open symbols) of 1 mM Ca^2+^. (**C**) Soluble α_2_ NTST or α_2_δ-1_S_ NTST (10 µg/ml) were coated onto 96-well plates and incubated with TSP-4 (1,000 nM). Binding assays (**A,C**) were carried out in the presence of 1 mM Ca^2+^ and bound proteins were detected with the corresponding TSP-specific antibody/antiserum (see Supplementary Table S2). Specific binding in (**A,C**) was calculated by subtracting OD values of non-specific binding from those of total binding. Data of total and non-specific binding were used to calculate *K*_D_ and B_max_ in (**B**), with the linear dependence of the non-specific signal on the TSP-4 concentration ensuring the absence of perturbations of the assay system. Data represent mean values ± SEM of 3 independent measurements performed in duplicates or triplicates. Statistical analysis in (**C**) was done using unpaired two-tailed Student’s t-test (***P = 0.0003).

First, the purified recombinant full-length TSPs (soluble) were examined for their direct interaction with immobilised α_2_δ-1_S_ NTST in an ELISA-style ligand binding assay, that was validated using the model interaction of COMP and matrilin-3 proteins^45^ (Supplementary Fig. S4). Preliminary experiments with TSP concentrations up to ~500 nM had demonstrated that out of the three TSPs generated in this study, only TSP-4 was able to directly interact with α_2_δ-1_S_ NTST (data not shown). To rule out the possibility of a very low binding affinity of TSP-2 and COMP towards α_2_δ-1, we finally utilised a comparably high concentration (1,000 nM) of all TSP proteins in the same ELISA which confirmed our preliminary data (Fig. 3A). Therefore, we decided to focus on the interaction of TSP-4 with α_2_δ-1_S_ and study it more in detail. Notably, we detected comparable binding signals of TSP-4 to either α_2_δ-1_S_ NTST or α_2_δ-1_S_ CTST variants in a preliminary experiment (data not shown) and hence we chose to proceed with one of the two variants, namely, α_2_δ-1_S_ NTST in our binding assays. Titration of immobilised α_2_δ-1_S_ NTST with increasing concentrations of TSP-4 (11-1,505 nM) showed saturable binding with an apparent *K*_D_ value of about 200 nM. Since Ca^2+^ binding is associated with major conformational changes and structural rearrangements of both TSPs^46,47^ and the metal ion-dependent adhesion site (MIDAS) motif of α_2_δ-1^17,48^, we investigated the effect of Ca^2+^ on TSP-4/α_2_δ-1_S_ NTST interaction. Our data show that the apparent *K*_D_ value was slightly, but not significantly, decreased in presence of 1 mM Ca^2+^ (Table 2, Fig. 3B). Similarly, the maximum binding value (B_max_, unitless) showed a small, statistically non-significant increase in presence of 1 mM Ca^2+^ (Table 2, Fig. 3B). These results indicate that the TSP-4/α_2_δ-1_S_ NTST interaction is not sensitive to Ca^2+^ changes. Next, the ability of TSP-4 to directly interact with the α_2_ fragment of α_2_δ-1 was analysed. Here, we observed a two-fold, statistically significant increase in the binding signal of a single concentration of TSP-4 (1,000 nM) when using immobilised α_2_ NTST instead of α_2_δ-1_S_ NTST (Fig. 3C). These results indicate the localisation of the TSP-4 binding site(s) within the α_2_ region of α_2_δ-1 in agreement with data obtained by Eroglu *et al*.^7^. The enhanced binding signal of TSP-4 to α_2_ NTST is suggestive of a more favourable conformation of α_2_ NTST which is more accessible for TSP-4 binding, as compared to the non-proteolytically cleaved α_2_δ-1_S_ NTST.

**Table 2:** Binding parameters (*K*_D_, B_max_) for the interaction of TSP-4 with α_2_δ-1_S_ NTST.

Assay buffer	*K*_D_ (nM)	B_max_
TBS	198 ± 49	0.579 ± 0.066
TBS + 1 mM Ca^2+^	153 ± 46	0.677 ± 0.054

Data represent mean values ± SEM of 3 independent experiments, each performed in duplicate. Statistical analysis by an unpaired two-tailed Student’s t-test showed no significant difference for *K*_D_ (apparent dissociation constant, *P* = 0.5381) and B_max_ (maximum binding, unitless, *P* = 0.3138) obtained in the absence and presence of Ca^2+^.

**Figure 4:**
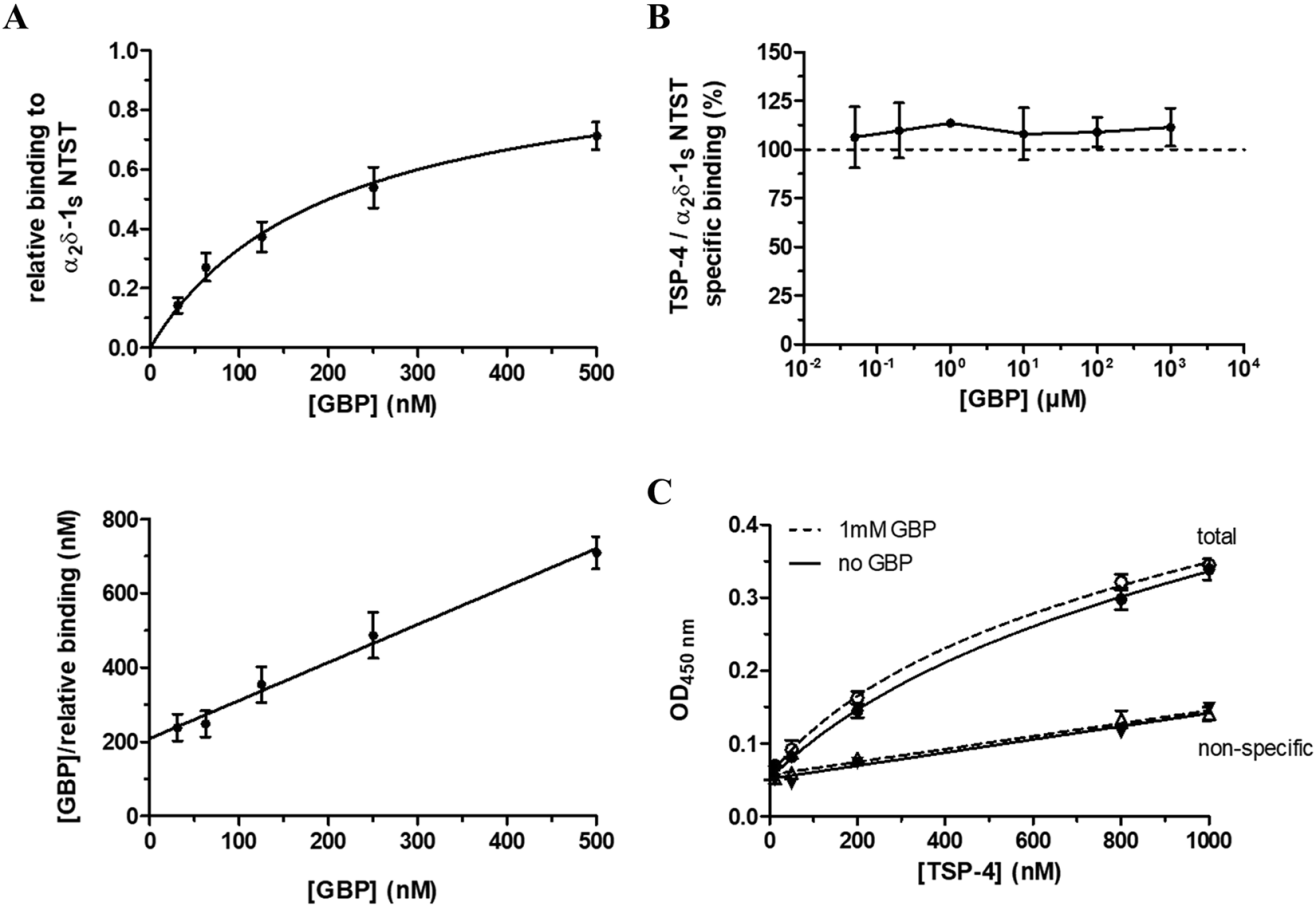
GBP does not directly interfere with the binding of TSP-4 to α_2_δ-1_S_ NTST in an ELISA-style ligand binding assay. (**A**) Surface plasmon resonance (SPR) measurements of the binding of GBP to recombinant α_2_δ-1_S_ NTST. The protein (10–15 µg/ml) was directly immobilised to CM5 sensor chips and GBP (31.25–500 nM) in PBS buffer, pH 7.4 containing 0.05% Tween 20 was passed over the chip at a flow rate of 30 μl/min. Shown are data of the relative GBP binding to α_2_δ-1_S_ NTST (Top) obtained from single cycle kinetics protocol (mean values ± SEM of 4 independent experiments, Fig. S3). Each experiment was analysed by non-linear regression according to the equation RU/RU_max_ = [GBP]/(*K*_D_ + [GBP]), where the ratio of the binding response and the maximum binding response, RU/RU_max,_ represents the relative binding at a given GBP concentration, [GBP], and *K*_D_ is the dissociation constant of the two interaction partners. Data analysis yielded a value of *K*_D_ = 219 ± 47 nM (mean value ± SEM, *n* = 4), with the linear shape of the Hanes-Woolf transformation (Bottom) showing equimolar binding of the two interaction partners. For the ELISA-style assay, the α_2_δ-1_S_ NTST (10 µg/ml) protein was coated onto 96-well plates and incubated with either (**B**) TSP-4 (1,000 nM) in the absence and presence of increasing concentrations of GBP (0.05–1,000 µM), or (**C**) increasing concentrations of TSP-4 (12.5–1,000 nM) in the absence (full symbols) and presence (open symbols) of GBP (1,000 µM). The assay was carried out in the presence of 2 mM Mg^2+^ and bound TSP-4 was detected with TSP-4-specific antiserum. Specific binding was calculated by subtracting OD values of non-specific binding (triangles) from those of total binding (circles). Data in (**B**) and (**C**) represent mean values ± SEM of 2 to 3 independent experiments, each performed in duplicate. In (**B**) the OD values for specific binding of TSP-4 in the presence of GBP (0.05–1,000 µM) were normalised to those in the absence of GBP.

To investigate the effect of the known α_2_δ-1 ligand GBP^20^ on the observed TSP-4/α_2_δ-1_S_ interaction, we checked for the ability of the recombinantly expressed mutant α_2_δ-1_S_ NTST to bind GBP with high affinity (*K*_D_ = 219 nM) using a label-free surface plasmon resonance (SPR) assay (Fig. 4A, Supplementary Fig. S3). Our results are in agreement with the binding data for ^3^H-GBP to membrane preparations of heterologously expressed human α_2_δ-1 (*K*_D_ range of 140–175 nM)^38^. Various GBP concentrations (0.05–1,000 µM) were used in the TSP-4 /α_2_δ-1_S_ NTST binding assay to interfere with this protein-protein interaction but did not show inhibitory effects (Fig. 4B). To rule out the possibility that the GBP binding pocket might not be accessible after immobilisation of α_2_δ-1_S_ NTST on the ELISA microplate, we had coated the wells with α_2_δ-1_S_ NTST after pre-incubating the protein with GBP. In addition, GBP had been supplemented to the liquid phase during blocking and incubation with TSP-4 ensuring availability of a sufficient number of GBP molecules to α_2_δ-1_S_ NTST and preventing dissociation of bound GBP during the experiment. In a further experiment, the binding of various concentrations of TSP-4 (12.5–1,000 nM) to α_2_δ-1_S_ NTST was not affected by the highest GBP concentration (1,000 µM) investigated (Fig. 4C). Together, these data suggest that GBP alone is not sufficient to disrupt the interaction of TSP-4 with α_2_δ-1 on a molecular level.

### Fluorescent A555-TSP-4 does not bind to membrane-bound α_2_δ-1 in a cell-based binding assay

**Figure 5:**
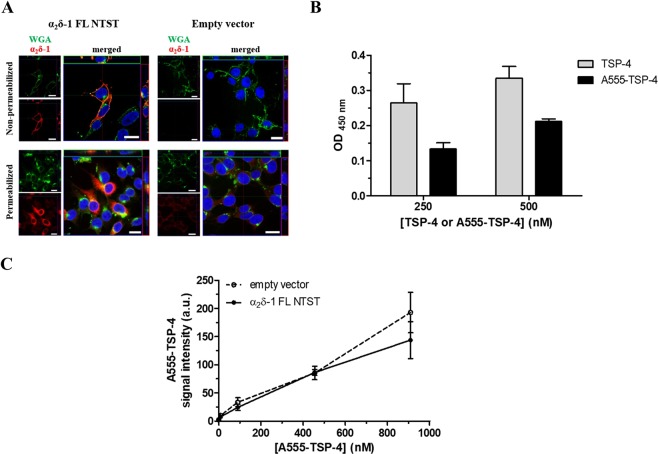
Fluorescent A555-TSP-4 protein does not bind to membrane-bound α_2_δ-1 in HEK293-EBNA cells. (**A**) Representative confocal images of the immunocytochemical detection of membrane-localised α_2_δ-1 FL NTST over-expressed in HEK293-EBNA cells. Cells were transfected with either empty vector (right column) or vector encoding full-length α_2_δ-1 containing an N-terminal strep II-tag (α_2_δ-1 FL NTST, left column). Cells were stained either without permeabilisation (upper row) or after membrane permeabilisation (lower row). Signals of WGA conjugated with Alexa Fluor 633 (green) and α_2_δ-1 (red) are shown individually in the small images; merged signals are shown in the large images. DAPI was used to visualise the nucleus (blue). Images show top view as well as upper-side (green box) and right-side (red box) views of a single slice of scanning near the middle of cells. Scale bar is 20 µm for all images. (**B**) Binding of A555-TSP-4 or TSP-4 to α_2_δ-1_S_ NTST analysed by an ELISA-style ligand binding assay. α_2_δ-1_S_ NTST (20 µg/ml) was coated onto 96-well plates and incubated with either TSP-4 or A555-TSP-4 at two different concentrations (250 and 500 nM) for each protein. Specific binding was calculated by subtracting OD values of non-specific binding from those of total binding. Data represent mean ± SEM of 2 independent measurements, each performed in duplicate. OD values for specific binding of TSP-4 and A555-TSP-4 were subjected to unpaired Student’s t-test. No significant difference was found (*P* > 0.05) for each of the two concentrations of both TSP-4 species used. (**C**) Cells transfected with either empty vector or vector encoding α_2_δ-1 FL NTST were incubated with increasing concentrations of A555-TSP-4 (final concentration: 9–909 nM) in 96-well imaging plates. Control wells received the same volume of dilution medium (40 µl) without A555-TSP-4. Average A555-TSP-4 fluorescent signal intensities from wells containing cells transfected with α_2_δ-1 FL NTST encoding vector or empty vector (±SEM of 3 independent experiments, each performed in triplicate) were plotted versus the A555-TSP-4 concentration.

To examine the interaction between TSP-4 and full-length membrane-bound α_2_δ-1 in a cellular environment, HEK293-EBNA cells were transfected with either empty vector or vector encoding α_2_δ-1 FL NTST. Immunocytochemical analysis of cells transfected with α_2_δ-1 FL NTST revealed a marked increase in α_2_δ-1 immunoreactivity, confirming the over-expression of heterologous α_2_δ-1 (Fig. 5A, left column).

In cells immunostained without permeabilisation, a high degree of co-localisation of α_2_δ-1 and wheat germ agglutinin (WGA) used for labelling glycoproteins or glycolipids of the outer leaflet of the plasma membrane^49^ were observed, assuring the localisation of heterologous α_2_δ-1 in the plasma membrane (Fig. 5A, upper left image). The transfected cells were incubated with increasing concentrations of fluorescently labelled A555-TSP-4 (9–909 nM), the binding of which to α_2_δ-1_S_ NTST was found to be non-significantly lower than that of unlabelled TSP-4 in ELISA-style assay (Fig. 5B). Images of the stained cells were acquired using a High Content Screening microscope and the average A555-TSP-4 signal intensity was determined for each well. In addition, α_2_δ-1 FL NTST was visualised by immunostaining to analyse the co-localisation of A555-TSP-4 with α_2_δ-1 FL NTST. Results show low overall A555-TSP-4 signal intensity which increases upon increasing the concentrations of the fluorescent protein. However, no difference was observed when comparing α_2_δ-1 FL NTST- and empty vector-transfected cells (Fig. 5C). Furthermore, no co-localisation of A555-TSP-4 and α_2_δ-1 FL NTST signals was detected in these experiments (Supplementary Fig. S5). These results are in accordance with recently published data by Lana *et al*.^38^ showing no TSP-4 co-localisation with α_2_δ-1 on the cell surface of tsA-201 cells co-expressing both proteins or in mixed populations of cells transfected separately with either α_2_δ-1 or TSP-4.

## Discussion

All TSPs, i.e. TSPs 1–4 and COMP, were previously identified as synaptogenic proteins which, together with other astrocyte-derived factors, help to promote the formation of functional excitatory synapses in the CNS^6,7^. The α_2_δ-1 protein was demonstrated to be functionally involved in TSP-induced synaptogenesis by means of synaptic assays in retinal ganglion cells (RGCs)^7^, DRG/spinal cord primary neuron co-culture^8,9^, purified cortical neurons^10^ as well as in dorsal spinal cord of mice^34^. Biochemically, α_2_δ-1 was reported to interact in co-immunoprecipitation experiments with TSP-1, TSP-2 and TSP-4 from rat cerebral cortex^7^ as well as with TSP-4 from rodent spinal cord^34^. Similarly, a TSP-2 fragment containing all three EGF-like repeats, the calcium-binding repeats, and the C-terminal globular domain was co-purified with full-length α_2_δ-1 or its protein-binding VWA domain after co-expression in HEK293 cells^7^. Recently, Park *et al*.^9,34^ demonstrated for the first time a direct molecular interaction between α_2_δ-1 and recombinant full-length TSP-4 or its fragments containing EGF-like or coiled coil domains. In the present study, we investigated the biochemical properties of the direct TSP-4/α_2_δ-1 interaction. Furthermore, it was of importance to know whether other members of the TSP protein family are also able to directly bind to α_2_δ-1 in an analogous manner to that of TSP-4. Our data demonstrated that only full-length TSP-4, but not TSP-2 or COMP, is able to directly interact with immobilised soluble α_2_δ-1 variant (α_2_δ-1_S_ NTST) in an ELISA-style ligand binding assay (Fig. 3A), indicating the specificity of this protein-protein interaction. This observation is in direct contrast to that of Eroglu *et al*.^7^ (see above). Nevertheless, TSP-4 is remarkably the only isoform of TSP proteins reported so far to be implicated in neuropathic and joint-mediated chronic pain in rodents along with neuronal α_2_δ-1^8,9,31,34,35^. During the processes resulting in such pain, both TSP-4 and α_2_δ-1 are up-regulated on the protein level and temporally correlate with the development of behavioural hypersensitivity in the respective animal models (for review see ref.^50^). In contrast, TSP-1/-2, though previously shown to be up-regulated after ischemic brain injury in rodents^4,51^ and promoting the subsequent synaptic recovery^51^, are not dysregulated on the protein level in dorsal spinal cord after spinal nerve ligation in mice, even when behavioural hypersensitivity was evident in these animals. This observation led Kim *et al*.^31^ to rule out the possibility of the involvement of these two astrocyte-secreted proteins in mediating TSP/α_2_δ-1-induced neuropathic pain. With regards to COMP, it has been reported to be expressed in several tissues including skeletal muscle, tendon, and cartilage. In the latter tissue, COMP is known to be mainly involved in chondrocyte differentiation, attachment, and cartilage extracellular matrix assembly^52–55^, with mutations in COMP being associated with pseudoachondroplasia^56,57^. COMP in skeletal muscle, tendons, and perichondrium can be theoretically in contact with nerve terminals containing α_2_δ-1. In addition, COMP was shown to have synaptogenic potential in RGCs (as discussed) and shares a high degree of both overall sequence identity (~70%) and structural similarity (Fig. 1A) with TSP-4. That is why we investigated COMP as a potential interaction partner of α_2_δ-1 in our binding studies. Nevertheless, COMP is, in contrast to TSP-4, neither abundant in neurons and astrocytes nor it is known to be dysregulated in neuropathic pain states. The fact that, beside TSP-4, all other TSPs were previously found to induce synaptogenesis through a mechanism involving neuronal α_2_δ-1^7^ may refer to other cellular factors or scaffold proteins required to mediate their interaction with α_2_δ-1 indirectly. Furthermore, it is tempting to speculate that TSP-induced synaptogenesis might be of little relevance to neuropathic pain development since, as previously mentioned, TSP-4 is the only member of TSP family found to be up-regulated in dorsal spinal cord following nerve injury. Indeed, a recent study shows that an enhanced presynaptic NMDA receptor activity, rather than synaptogenesis, is responsible for maintaining the increased synaptic excitatory transmission in dorsal spinal cord leading to chronic pain states following nerve injury in mice^58^.

In further experiments, we observed a significantly increased binding of TSP-4 to α_2_ NTST when compared to α_2_δ-1_S_ NTST (Fig. 3C), confirming previous data by Eroglu *et al*.^7^ demonstrating the TSP-4 binding site to be localised within the α_2_ region of α_2_δ-1 (VWA domain). The observed enhancement in binding towards α_2_ might be attributed to more exposed TSP-4 binding motif(s) in the immobilised α_2_ NTST compared to the non-proteolytically processed α_2_δ-1_S_ NTST. This result is in agreement with recent findings from Lana *et al*.^38^ where wild-type α_2_δ-1 was very weakly co-immunoprecipitated with TSP-4, but no co-immunoprecipitation of the mutant α_2_δ-1 (MIDAS^AAA^) with TSP-4 was detected in lysates of co-transfected tsA-201 cells. In addition, the binding to α_2_ NTST seems again to be TSP-4-specific since negligible binding signals were detected when equimolar concentrations of either COMP or a truncated TSP-4 fragment were utilized in a pilot experiment (data not shown). Although our results are supported by reported data, we cannot rule out the possibility that the enhanced TSP-4 binding signal is due to improperly expressed α_2_ NTST since unpaired cysteine residues, normally involved in the formation of disulphide bridges with other cysteine residues in the deleted regions of wild-type α_2_δ-1^59^, become available. It is worth mentioning here that a very high tendency for multimerisation was observed for a recombinant VWA domain of α_2_δ-1 generated in this study (data not shown) due to the formation of intermolecular disulphide bonds. Therefore, it might be appropriate to consider the expression of α_2_ and VWA fragments in which the unpaired cysteines are replaced by other isosteric residues (e.g. serine) for future binding studies.

One of the reported small molecules capable of interfering with the TSP/α_2_δ-1 interaction is GBP, an approved analgesic against neuropathic pain^60–62^ and a known ligand of α_2_δ-1^20^. Biochemically, co-immunoprecipitation experiments showed that the interaction between a truncated TSP-2 fragment and α_2_δ-1 FLAG in a co-culture of two populations of HEK293 cells was diminished in the presence of GBP^7^. In addition, as previously mentioned, TSP-4 modestly but significantly reduces the binding affinity of ^3^H-GBP to α_2_δ-1, suggesting rather an allosteric than a pure competitive mode of inhibition^38^. Furthermore, *in vivo* data revealed the ability of GBP to block TSP-4-induced neuronal sensitisation and behavioural hypersensitivity as well as changes in Ca^2+^ currents and intracellular Ca^2+^ transients after injuries to peripheral nerves or facet-joint in rodents^8,9,34,35,63^. Similarly, several studies in neuropathic pain models demonstrated the ability of GBP to inhibit α_2_δ-1-induced^26^ or TSP-induced^7,8,34,35^ synaptogenesis. Most recently, GBP was also shown to inhibit TSP-2-induced synapse formation in purified culture of cortical neurons^10^. Despite the multidimensional evidence of GBP interference with TSP/α_2_δ-1 interaction, a direct GBP inhibition of this interaction on the molecular level has never been investigated before, to our knowledge. In the current study, we did not observe any inhibition of the direct TSP-4/α_2_δ-1_S_ NTST interaction in the presence of increasing concentrations (up to 1 mM) of GBP (Fig. 4B). Furthermore, the highest GBP concentration used (1 mM) did not shift the TSP-4/α_2_δ-1_S_ NTST binding curve (Fig. 4C). Although the utilised α_2_δ-1_S_ NTST was mostly expressed as uncleaved form of the protein (in agreement with the original work describing a similar porcine α_2_δ-1 mutant^40^), we were able to demonstrate the ability of this α_2_δ-1_S_ mutant to retain high affinity for GBP (Fig. 4A). For this purpose, a newly developed SPR-based binding assay suitable for detecting and quantifying the binding of small molecules to immobilized recombinant α_2_δ-1_S_ was used. This SPR assay has the advantage of being radiolabel-free and can easily be used to determine the binding kinetics unlike the previously used ^3^H-GBP binding assay^38,40,64,65^. Taken together, our data confirmed that the proteolytic cleavage of α_2_δ-1 is not crucial for the formation of the GBP binding pocket^40^. The complete lack of GBP inhibition towards the interaction of purified TSP-4 with α_2_δ-1_S_ NTST raises questions regarding the exact mechanism by which GBP can block the above-mentioned TSP-induced changes. It is possible that other unknown factors in the cellular environment are essential for GBP to interfere with α_2_δ-1/TSP-4 interaction and thereby mediating the known GBP inhibitory effects. Another possible explanation based on the recent findings of Chen *et al*.^58,66^ is that the α_2_δ-1/NMDA receptor complex, rather than α_2_δ-1/TSP-4 binding, represents the molecular target of gabapentinoid drugs to alleviate neuropathic pain.

Our efforts were as well focused on the investigation of the TSP-4/α_2_δ-1 interaction in a cellular system to get closer to the physiological/pathological situation in the CNS. We over-expressed full-length α_2_δ-1 in HEK293-EBNA cells and demonstrated both its intracellular and plasma membrane localisation in transfected cells (Fig. 5A). Treatment with increasing concentrations (up to 909 nM) of fluorescently labelled A555-TSP-4, however, showed no differences in binding of the protein to α_2_δ-1 overexpressing cells when compared to control cells (Fig. 5C, Supplementary Fig. S5). Furthermore, fluorescent signals of A555-TSP-4 did not co-localise with those of immunostained α_2_δ-1 on the cells (Supplementary Fig. S5). The observed loss of binding cannot be attributed to an impairment of the interaction of the two proteins by the fluorescent label of TSP-4 since the fluorescent protein was generated with a minimal dye-to-protein molar ratio and showing substantial α_2_δ-1_S_ NTST binding in the ELISA-style assay (Fig. 5B). A possible explanation could instead be arising from the weak interaction of the two proteins under the conditions of the cell-based assay, unlike the ELISA-style assay. This means that very high local concentrations of TSP-4 in the proximity of cell-surface α_2_δ-1 would be required to enable the detection of their interaction by simulating the pathological situation (e.g. dramatic up-regulation following nerve injury). This could not, however, be achieved with the range of A555-TSP-4 concentrations (up to 909 nM) used in the assay. In our experiments, we over-expressed α_2_δ-1 in HEK293-EBNA cells without co-expression of α_1_ subunit, which interacts intracellularly with α_2_δ-1 before trafficking of the complex to the cell surface^67^. However, we assume that the lack of α_1_ subunit did not hamper the putative binding of TSP-4 to α_2_δ-1 on the cell surface. This assumption is based on recent data showing the ability of wild-type α_2_δ-1, expressed in HEK293 cells without co-expression of α_1_ subunits, to be efficiently transported to the cell surface and thereby become accessible to extracellular ligands like TSP^10^. Functionally, this α_2_δ-1 over-expression construct alone was able to rescue synapses in cortical organotypic slices from α_2_δ-1 knockout mice. This effect is found to be mediated through activation of the small Rho GTPase Ras-related C3 botulinum toxin substrate 1 (Rac1) and is independent of α_1_ subunits of the postsynaptic L-type calcium channels Ca_*V*_1.2 and Ca_*V*_ 1.3^10^.

The α_2_δ subunits are thought to promote membrane trafficking of the pore subunits of voltage-gated calcium channels^17^ and α_2_δ-1-driven allodynia in mice can be reversed by blockers of voltage-gated calcium channels like ω-conotoxin GVIA^68^. However, other findings suggest that the maladaptive changes contributing to chronic pain in rodents following nerve injuries and resulting from the interaction of dysregulated TSP-4 with α_2_δ-1 are partially independent of the role of the latter protein in regulating voltage-gated calcium channels’ trafficking and function^50^.

As previously mentioned, our data align with those of Lana *et al*.^38^ who reported no interaction of secreted TSP-4 with membrane-localised α_2_δ-1 on tsA-201 cells when subjected to immunocytochemical analysis. On the other hand, the same study showed weak intracellular interaction of both proteins in co-immunoprecipitation experiments from cells over-expressing both proteins^38^. It has therefore been postulated that the weak TSP-4/α_2_δ-1 interaction may occur in an intracellular compartment rather than on the cell surface^38,69^. This postulation is in contrast to the previous data showing synaptogenic effect of secreted TSPs which is mediated by neuronal α_2_δ-1 thought to be located either pre-^8^ or post-^7,10^ synaptically. To our knowledge, there are no data so far showing the co-localisation of TSP(-4) and α_2_δ-1 in neuronal cell cultures or spinal cord tissue where TSP-induced changes (e.g. synaptogenesis) were demonstrated. To reveal the exact cellular localisation of TSP-4/α_2_δ-1 interaction it would be very helpful to simultaneously analyse co-immunostained TSP-4 and α_2_δ-1 proteins in cultures of neurons utilized in *in vitro* synapse assays^6,7,10^.

In summary, our results provide substantial *in vitro* biochemical evidence for a direct and specific Ca^2+^-insensitive TSP-4/α_2_δ-1 interaction which is rather weak. Importantly, GBP does not inhibit this interaction on a molecular level, indicating the possible involvement of other unknown factors or targets in mediating GBP inhibitory effects in neuropathic pain.

We, therefore, need to understand the exact and complete molecular mechanism of the TSP/α_2_δ‐1 interaction to really be able to design appropriate small molecule modulators - rather than being left to use and optimize the enigmatic properties of the serendipitously discovered gabapentinoid action.

## Materials and Methods

### Cloning, expression and purification of recombinant proteins

The plasmids encoding full-length murine TSP-2 (TSP-2, accession no. of the translated protein product AAH53702.1), full-length rat COMP (COMP, accession no. of the translated protein product EDL90681.1), and full-length rat TSP-4 (TSP-4, accession no. of the translated protein product NP_058829.1) were generated as described earlier for COMP^70^. The cDNAs encoding human α_2_δ‐1 soluble variant^40^ with an N-terminal or a C-terminal double strep II-tag (α_2_δ‐1_S_ NTST and α_2_δ‐1_S_ CTST, respectively) and its α_2_ peptide chain with an N-terminal double strep II-tag (α_2_ NTST) were amplified following the same protocol from a pIRESpuro3-α_2_δ‐1 vector, originally generated from human α_2_δ‐1 FL cDNA^71^ (α_2_δ‐1, accession no. of translated protein product XP_005250627.1). All PCR products were lacking the natural signal peptide sequences and harboured a *Nhe*I restriction site at the 5′-end and a *Xho*I or *Bam*HI site at the 3′-end. After digestion with *Nhe*I and either *Xho*I or *Bam*HI, the amplified cDNAs were cloned into the modified episomal expression vector pCEP-Pu-double strep II-tag (N- or C-terminal)^72^ in-frame with the 5′ sequence of the BM-40 signal peptide and confirmed by Sanger nucleotide sequencing. Here, the sequences of the double strep II-tag and the thrombin cleavage site were directly located either at the 5′- or the 3′-end of the inserted cDNA sequences.

The recombinant plasmids carrying cDNAs encoding full-length proteins or truncated fragments were transfected into human embryonic kidney 293/Epstein-Barr virus nuclear antigen cells (HEK293-EBNA, Invitrogen) using TurboFect transfection reagent (Thermo Fischer Scientific). Transfected cells were cultured in DMEM medium supplemented with 10% FCS (Gibco), penicillin (1000 U/ml)/streptomycin (1000 µg/ml), and puromycin (3 µg/ml, Gold Biotechnology) for positive selection and were incubated at 37 °C/5% CO_2_ to allow growth to 100% confluency as adherent monolayers in cell culture triple-flasks (Nunc^TM^, Thermo Fisher Scientific). Cell culture supernatants containing the secreted target proteins were collected and supplemented with phenylmethylsulfonyl fluoride (1 mM, Applichem) before passing over self-packed streptactin-sepharose columns (0.5 ml, IBA). The recombinant double strep II-tagged proteins were eluted with phosphate-buffered saline (PBS), pH 7.4 containing D-desthiobiotin (2.5 mM, Sigma), concentrated with Amicon ultra centrifugal filter units with molecular weight cutoff of 100 kDa for all recombinant proteins (Millipore), aliquoted after addition of glycerol (10% (v/v)) and stored at −80 °C until use.

### Coomassie stained gels and immunoblotting analysis

For protein analysis by Coomassie staining, the purified TSPs were separated on 0.5% (w/v) agarose/3% (w/v) polyacrylamide composite gels^73^ without prior DTT treatment, while α_2_δ-1_S_ NTST and α_2_ NTST variants were separated using conventional SDS-PAGE on 7% gels in presence and absence of DTT. Gels were stained overnight with colloidal Coomassie staining solution followed by destaining for 1–2 hours in a solution of ethanol (10% v/v) and o-phosphoric acid (2% v/v). All purified proteins were subjected to immunoblotting analysis by separating the proteins using SDS-PAGE under non-reducing and reducing conditions on 4–15% gradient gels for TSPs, 10% gels for α_2_δ‐1_S_ NTST and α_2_δ‐1_S_ CTST (before and after digestion with thrombin), and 7% gel for α_2_ NTST. Proteins were then transferred onto PVDF membranes by blotting overnight at 4 °C and blocked with 5% skimmed milk in Tris-buffered saline (TBS), pH 7.4 for 1 h at RT. The blocked membranes were incubated with antibodies recognizing the (double) strep II-tag or specific epitopes of the proteins for 1 h at RT, followed by incubation with the corresponding horseradish peroxidase-conjugated secondary antibodies (see list of antibodies used in Supplementary Table S2) for 1 h at RT. Protein bands were visualised using the Odyssey Fc Imaging system (LI-COR Biosciences).

### Mass spectrometry

The masses of the purified recombinant α_2_δ‐1_S_ NTST and TSPs were determined by MALDI-TOF mass spectrometry performed in the bioanalytical laboratory of the Centre for Molecular Medicine Cologne (CMMC) using a similar protocol to that described by Klatt *et al*.^74^. Briefly, TSPs and α_2_δ‐1_S_ NTST were first incubated with DTT (10 mM) in PBS overnight at 4 °C with additional sample of α_2_δ‐1_S_ NTST being treated similarly but without DTT. Next, samples were desalted with micro Zeba Spin desalting columns (Pierce Biotechnology, Thermo Fisher Scientific) equilibrated in trifluoroacetic acid (0.1% (v/v)) in ultrapure water. 5 µl of the eluate were mixed with 5 µl of a saturated solution of sinapinic acid (Bruker) in acetonitrile (50% (v/v)), trifluoroacetic acid (0.1% (v/v)), and 1 µl of the mixture was applied onto a MTP 384 polished steel target (Bruker). MALDI spectra were acquired with an Ultraflextreme MALDI-TOF/TOF instrument (Bruker) operated in linear mode. The monomer ([M + H]^+^, [M + 2 H]^2+^) and dimer ([2 M + H]^+^) peaks of bovine serum albumin (BSA, Applichem) were used for external calibration of the spectra. Analysis of the MS spectra was done with FlexAnalysis software (Bruker).

### Fluorescence labelling of TSP-4

TSP-4 was labelled with Alexa Fluor 555 succinimidyl ester (Life Technologies) according to manufacturer’s instructions. Briefly, buffer exchange of protein samples to 100 mM NaHCO_3,_ 100 mM NaCl (pH 8.3) was performed using Amicon ultra centrifugal filter unit (Millipore). Protein sample was then incubated with a five-fold amount of Alexa Fluor 555 succinimidyl ester (stock dissolved at 10 mg/ml in dry dimethylformamide) for 1 h at 25 °C followed by addition of 10% (v/v) hydroxylamine (1.5 M), pH 8.5, and incubation of the hydroxylamine-containing reaction mixture for 1 h to terminate the reaction. Free dye was then separated using gel filtration by loading the protein/dye reaction mixture onto a 15-ml self-packed Sephadex G-25 (Sigma) column and elution with PBS, pH 7.4. Fractions containing labelled protein eluted in the void volume were pooled, mixed with glycerol 10% (v/v) and stored at −80 °C until use.

### ELISA-style ligand binding assay

Purified α_2_δ‐1_S_ NTST (10 or 20 µg/ml) or α_2_ NTST (10 µg/ml) in coating buffer (TBS, pH 7.4 with or without 1 mM Ca^2+^ or with 2 mM Mg^2+^ for experiments with GBP) were coated overnight at RT onto 96-well plates (Nunc Maxisorb) to obtain 0.5–1 µg protein/well for determination of values of total binding. Values of non-specific binding were obtained by adding coating buffer to the wells and proceeding similarly. After a three-cycle wash with TBS/0.05% Tween 20, pH 7.4 using a Tecan plate washer HydroFlex, wells were blocked for 2 h at RT with 5% skimmed milk in TBS, pH 7.4. Full-length TSPs and A555-TSP-4 were diluted in 5% skimmed milk in TBS, pH 7.4. TSP-4 final concentrations of either 11-1,505 nM or 12.5–1,000 nM were utilised for titration experiments in presence or absence of Ca^2+^ (1 mM) or in presence of GBP (1,000 µM) and Mg^2+^ (2 mM), respectively, while other TSPs and A555-TSP-4 were applied at final concentrations of 250, 500 or 1,000 nM for single point determinations. All protein ligands were incubated in the corresponding wells for 2 h at RT. Experiments investigating the influence of GBP on the interaction of α_2_δ‐1_S_ NTST and TSP-4 were done by adding the respective amount of GBP (Sigma-Aldrich, #G154, 100 mM stock in water, final concentration: 0.05–1,000 µM) during coating, blocking and incubation with TSP-4. After a three-cycle wash with TBS/0.05% Tween 20, pH 7.4, bound ligands were incubated with TSP-specific antibodies followed by corresponding secondary antibodies conjugated to horseradish peroxidase (Supplementary Table S2). Quantification of bound TSPs was achieved by measuring horseradish peroxidase (HRP) activity using tetramethylbenzidine (62.5 µg/ml, Roth) and hydrogen peroxide (0.00525% (v/v)) as substrate (50 µl substrate solution/well). Absorbance was measured at 450 nm on a Biotek Synergy 2 multimode microplate reader with the software Gen 5 version 1.11.5 after stopping the enzymatic reaction by adding 50 µl/well of sulfuric acid (10% (v/v)). Data of total and non-specific binding were used to calculate apparent *K*_D_ values with GraphPad Prism v5.04 for Windows (GraphPad Software, San Diego, CA, USA).

### Surface Plasmon Resonance (SPR)

The GBP-α_2_δ‐1_S_ NTST binding experiments were carried out using BIAcore T200 and CM5 Sensor Chips (GE Healthcare) at 25 °C. The recombinant α_2_δ‐1_S_ NTST protein was diluted in immobilisation buffer (sodium acetate, 10 mM, pH 5) at 10–15 µg/ml and injected at a flow rate of 10 µl/min for 7 min to be directly coupled to the dextran matrix of a CM5 sensor chip flow cells using amine coupling kit as described before^75^. Excess reactive esters were quenched by injection of 1 M ethanolamine-hydrochloride, pH 8.5. The binding assays were performed using degassed PBS buffer, pH 7.4 containing 0.05% Tween 20 as running buffer. A serial dilution of GBP at concentrations of 31.25–500 nM was prepared in the running buffer and injected at a flow rate of 30 μl/min over the α_2_δ‐1_S_ NTST-coated and reference flow cells in a single cycle kinetics protocol. Non-specific binding signals of GBP observed in the flow cell with no immobilized protein were subtracted from all sensograms using BIAevaluation software (version 2.0, GE Healthcare Sciences). Relative binding of GBP to α_2_δ‐1_S_ NTST was plotted versus the GBP concentration with equimolar interaction of the binding partners being verified by means of a Hanes-Woolf transformation. Data plotting and analysis was done with GraphPad Prism v5.04 for Windows (GraphPad Software, San Diego, CA, USA).

### Immunostaining of HEK293-EBNA cells

HEK293-EBNA cells transfected with α_2_δ-1 FL NTST encoding vector or empty vector using Viromer Red transfection reagent (Lipocalyx) were plated in flat-bottom wells of poly-l-lysine-coated µ-slides (8-well, ibidi GmbH) and grown in appropriate DMEM medium supplemented with 3 µg/ml puromycin for positive selection of transfected cells. The cells were incubated at 37 °C/5% CO_2_ until they reached 60–70% confluency and then immunostained. Briefly, cells in all wells on the µ-slide were washed 2x with PBS, immediately fixed with 4% paraformaldehyde in PBS for 5 minutes, and subsequently washed again 2x with PBS (all done at RT). Cells were then either permeabilised by addition of 0.2% Triton X-100 in PBS for 15 min at RT or left with no permeabilization followed by a similar washing step of all cells.

To reduce non-specific staining, cells were incubated with 5% donkey serum (Sigma-Aldrich, D9663) in PBS for 60 min at RT. Subsequently, cells in all wells were incubated with anti-dihydropyridine receptor antibody (α_2_ subunit, Sigma, D219), diluted at 1:100 in 1% donkey serum/PBS overnight at 4 °C, followed by incubation with donkey anti-mouse IgG-Alexa Fluor^®^ 568 antibody (1:300) in 1% donkey serum/PBS containing DAPI for 1 h at RT. Next, cells were washed with PBS and incubated for 15 min with fluorescent wheat germ agglutinin (WGA)-Alexa Fluor 633 conjugate (Life Technologies, #W21404) diluted at 1:200 in PBS, for co-staining of plasma membrane. Unbound WGA was subsequently removed through a double wash step with PBS. Finally, cells were mounted in DABCO antifade solution (Carl Roth) to reduce photobleaching. Cells were examined on a confocal laser scanning microscope Zeiss LSM880 using 63× oil-immersion objective. The confocal optical sections were adjusted to 0.4–0.6 μm. Images were processed using Zen 2.3 lite software and assembled using Adobe Photoshop CS5.1 v.12.0.4 (Adobe Systems Inc., San Jose, CA, USA).

### Cell-based protein binding assay

HEK293-EBNA cells transfected with α_2_δ-1 FL NTST encoding vector or empty vector using TurboFect transfection reagent (Thermo Fischer Scientific) were suspended in DMEM cell culture medium supplemented with 10% FCS (Gibco), penicillin (1000 U/ml)/streptomycin (1000 µg/ml), and puromycin (3 µg/ml, Gold Biotechnology). The cells were seeded at 4,800–6,000 cells/well onto 96-well imaging plates (Greiner) after pre-coating with poly l-ornithine (0.1 mg/ml, Sigma) and were incubated overnight at 37 °C/5% CO_2_ prior to the day of experiment. On the day of experiment, the cell culture medium in the wells was exchanged with 40 µl of fluorescently labelled A555-TSP-4 in increasing concentrations (final concentration: 9–909 nM) diluted in cell culture medium. Control wells received the same volume of medium (40 µl) without A555-TSP-4. After incubation for 1 h at RT, cells in the wells were washed 3x with cell culture medium before fixation with 4% paraformaldehyde (Sigma) in PBS for 10 min at RT. Cells were then rinsed 3x with PBS (100 µl/well) followed by blocking for 1 h at RT with 50 µl normal goat serum blocking buffer containing 2% goat serum (Dianova), 1% bovine serum albumin, 0.1% Triton X-100 in PBS/0.05% Tween 20. One out of three-replicate wells for both control and A555-TSP-4-treated cells were then incubated with 30 µl of either mouse anti-dihydropyridine receptor (α_2_ subunit) antibody or mouse anti-strep II-tag antibody after dilution in PBS/1% BSA at 1:500 and 1:200, respectively, while the second and third replicate wells received the same volume (30 µl) of PBS/1% BSA without antibody. The plate was then left overnight at 4 °C. On the next day, cells were first rinsed 3x with PBS (with 10 min intervals) before addition of 50 µl/well of donkey anti-mouse IgG-Alexa Fluor^®^ 488 conjugated secondary antibody diluted at 1:1000 in PBS and containing DAPI (0.05 µg/ml, Molecular Probes) for nuclear staining. The plate was then left in the dark for 1 h at RT followed by rinsing the cells with PBS as described before. Finally, the wells were filled with 200 µl of PBS, sealed with aluminum sealing and stored at 4 °C until scanning. Images of the stained cells (1104 × 1104 pixels) were acquired using a Cellomics ArrayScan XTI microscope with a light-emitting diode light source using a 20× objective and analysed using the Cellomics software package v.6.6.0 (Thermo Scientific Cellomics HCS Studio). Cell nuclei were identified by DAPI staining according to the object identification parameters: size, 40–800 μm^2^; ratio of perimeter squared to 4π area, 1–3; length-to-width ratio, 1–5; average intensity, 500–5000; total intensity, 2 × 10^5^–1 × 10^7^. The cellular region of interest was defined by extending the nuclear region by maximally 10 μm and used to quantify fluorescence signals at desired wavelengths. Average signal intensity of A555-TSP-4 from three independent experiments was plotted against A555-TSP-4 concentrations.

## Supplementary information

Supplementary information

## Data Availability

The data generated and/or analysed during the current study are available from the corresponding author on reasonable request.
