# Supplementary material for: Direct, gabapentin-insensitive interaction of a soluble form of the calcium channel subunit α2δ-1 with thrombospondin-4
Source: Sci Rep. 2019 Nov 7;9:16272. doi: 10.1038/s41598-019-52655-y (PMC6838084; doi:10.1038/s41598-019-52655-y)
Supplement: Supplementary file 1 — Supplementary information [file 41598_2019_52655_MOESM1_ESM.pdf]

Supplementary information

Direct, gabapentin-insensitive interaction of a soluble form of the calcium channel subunit $\alpha_2\delta$-1 with thrombospondin-4

Ehab El-Awaad;[1,2] Galyna Pryymachuk;[3] Cora Fried;[1] Jan Matthes;[1] Jörg Isensee;[4] Tim Hucho;[4] Wolfram F. Neiss;[3] Mats Paulsson;[5,6] Stefan Herzig;[1,7] Frank Zaucke;[5,8] Markus Pietsch[1*]

[1] Institute II for Pharmacology, Centre of Pharmacology, Medical Faculty, University of Cologne, Gleueler Str. 24, D-50931 Cologne, Germany

[2] Department of Pharmacology, Faculty of Medicine, Assiut University, Assiut 71515, Egypt

[3] Department of Anatomy I, Medical Faculty, University of Cologne, Kerpener Str. 62, D-50937 Cologne, Germany

[4] Experimental Anaesthesiology and Pain Research, Department of Anaesthesiology and Intensive Care Medicine, Medical Faculty, University of Cologne, Robert-Koch-Str. 10, D-50931 Cologne, Germany

[5] Institute for Biochemistry II, Centre for Biochemistry, Medical Faculty, University of Cologne, Joseph-Stelzmann-Str. 52, D-50931 Cologne, Germany

[6] Centre for Molecular Medicine Cologne (CMMC), University of Cologne, Robert-Koch-Str. 21, D-50931 Cologne, Germany

[7] President of TH Köln, TH Köln (University of Applied Sciences), Claudiusstr. 1, D-50678, Cologne, Germany

[8] Dr. Rolf M. Schwiete Research Unit for Osteoarthritis, Orthopedic University Hospital, Friedrichsheim gGmbH, Marienburgstr. 2, D-60528, Frankfurt/Main, Germany

Supplementary Table S1. PCR primers (forward, fwd; reverse, rev) used for cloning of recombinant constructs and their nucleotide sequences. Introduced restriction sites were those of *Nhe*I (gctagc, fwd), *Bam*HI (ggatcc, rev) and *Xho*I (ctcgag, rev), respectively; inserted stop codons (TAA, TGA) are depicted in bold letters as reverse complimentary sequences.

**Protein name**	**Primers**	**Sequence 5′→3′**
**$\alpha_2\delta$-1 FL NTST**	P1 fwd	AAAgctagcGAGCCGTTCCCTTCG
	P2 rev	TTTggatcc**TCA**TAACAGGCGGTGTG
**$\alpha_2\delta$-1$_S$ NTST**	P1 fwd	AAAgctagcGAGCCGTTCCCTTCG
	P3 rev	AAActcgag**TTA**TCCAGAAACACCACCACAGTC
**$\alpha_2\delta$-1$_S$ CTST**	P4 fwd	AAAgctagcAGAGCCGTTCCCTTCG
	P5 rev	AAActcgagTCCAGAAACACCACCACAGTC
**α_2 NTST**	P1 fwd	AAAgctagcGAGCCGTTCCCTTCG
	P6 rev	TTTggatcc**TCA**CTCAAGGAGTCGTGG

Supplementary Table S2. List of primary and secondary antibodies used for ELISA, Western blot (WB) immunocytochemistry (ICC) and cell-based assay.

	Antibody	Clonality	Dilution	Product number/ Manufacturer or Reference	Application
primary antibodies	mouse anti-TSP-2	monoclonal	1:500	611150/BD Biosciences	WB, ELISA
	mouse anti-strep II-tag	monoclonal	1:500	2-1507-001/IBA	WB
	rabbit anti-human $\alpha_2\delta$-1	polyclonal	1:2000	OSC00098W/Osenses	WB
	mouse anti-dihydropyridine receptor (α_2 subunit)	monoclonal	1:100-1:500	D219/Sigma	ICC, cell-based assay
	guinea pig anti-rat TSP-4 antiserum	polyclonal	1:2000-1:8000	generated in the Institute for Biochemistry II, Centre for Biochemistry, Medical Faculty, University of Cologne[1-4]	WB, ELISA
	chicken anti-rat COMP antiserum	polyclonal	1:2000	AP1009/Immundiagnostik	WB, ELISA
secondary antibodies	rabbit anti-mouse IgG-HRP conjugated	polyclonal	1:1000	P0260/Dako	WB, ELISA
	swine anti-rabbit IgG-HRP conjugated	polyclonal	1:1000	P0399/Dako	WB
	rabbit anti-guinea pig IgG-HRP conjugated	polyclonal	1:2000	A5545/Sigma	WB, ELISA
	rabbit anti-chicken IgG-HRP conjugated	polyclonal	1:25000	303-035-003/Jackson ImmunoResearch	WB, ELISA
	donkey anti-mouse IgG-Alexa Fluor® 488 conjugated	polyclonal	1:1000	A21202/Life Technologies	Cell-based assay
	donkey anti-mouse IgG-Alexa Fluor® 568 conjugated	polyclonal	1:300	A10037/Life Technologies	ICC

Supplementary Fig. S1. Western blot analysis of purified recombinant proteins.

Immunoblot analysis of **(a)** three full-length TSP proteins, all carrying an N-terminal strep II-tag: TSP-2, TSP-4, and COMP; **(b)** soluble $\alpha_2\delta$-1 variants carrying either an N-terminal ($\alpha_2\delta$-1_S NTST) or a C-terminal ($\alpha_2\delta$-1_S CTST) strep II-tag and the product of the thrombin digestion of $\alpha_2\delta$-1_S CTST ($\alpha_2\delta$-1_S CTST + thrombin) (*The first and second lanes of the two immunoblots in the lower panel correspond to Fig. 2c). Proteins were separated under non-reducing (-DTT) or reducing (+DTT) conditions on 4-15% **(a)** or 10% **(b)** polyacrylamide gels, respectively. Proteins were detected with anti-TSP-2 antibody, TSP-4 and COMP antisera, rabbit anti-$\alpha_2\delta$-1, and mouse anti-strep II-tag. Secondary antibodies included the polyclonal rabbit anti-mouse IgG, anti-guinea pig IgG, anti-rabbit IgG, and anti-chicken IgG, all conjugated with horseradish peroxidase (see **Supplementary Table S2** for further information). Molecular weight standard (in kDa) indicated on the left was PageRuler Plus Prestained Protein Ladder (Thermo Fisher Scientific).

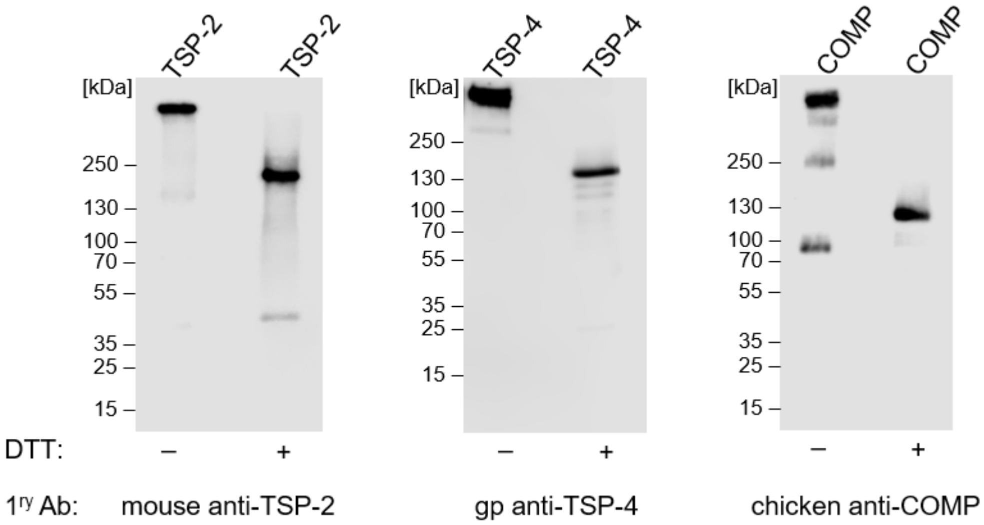

Fig. S1a.

The trimeric TSP-2 and the pentameric TSP-4 and COMP appear as high molecular weight bands in the absence of DTT, corresponding to the respective oligomeric proteins **(Fig. 2a and 2b, -DTT; Supplementary Fig. S1a, -DTT)**. However, faint bands with a lower *M*r were occasionally visible under non-reducing conditions for the three full-length TSPs using both the strep II-tag and specific antibodies **(Fig. 2b, -DTT; Supplementary Fig. S1a, -DTT)**, representing different oligomerisation states of the proteins as previously reported for COMP[5,6]. For TSP-2, an additional, distinct band is visible under both non-reducing (*M*r ~86 kDa) and reducing conditions (*M*r ~44 kDa), upon detection with strep II-tag antibody **(Fig. 2b)**, indicating possible minor degradation product of the protein from its N-terminus.

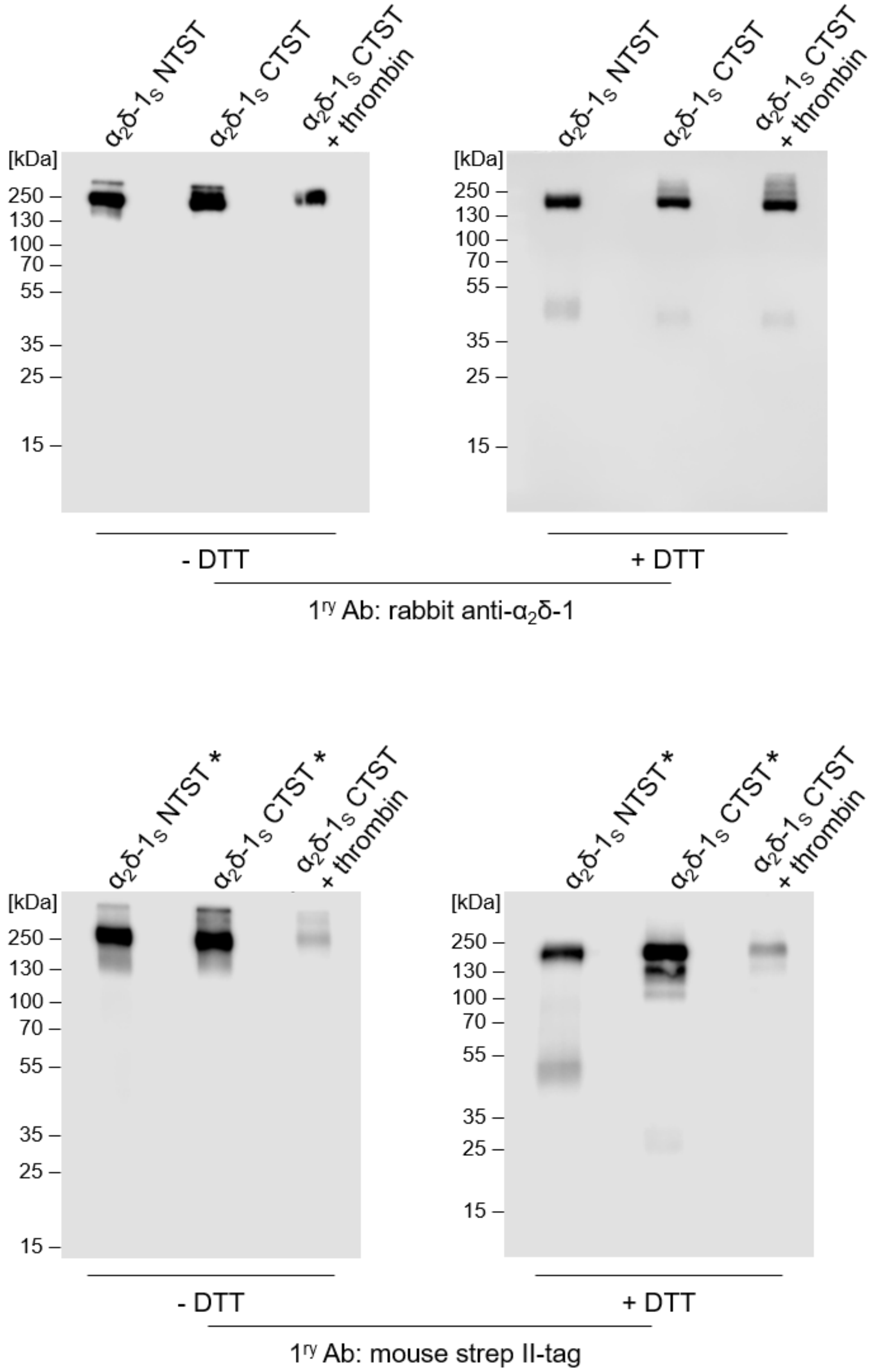

α2δ-1S NTST
α2δ-1S CTST
α2δ-1S CTST + thrombin
[kDa]
250
130
100
70
55
35
25
15
- DTT
+ DTT
1ry Ab: rabbit anti-α2δ-1
α2δ-1S NTST *
α2δ-1S CTST *
α2δ-1S CTST + thrombin
- DTT
+ DTT
1ry Ab: mouse strep II-tag

Fig. S1b.

Cleavage of the C-terminal double strep II-tag of $\alpha_2\delta$-1_S CTST by thrombin digestion resulted in weaker band upon detection using strep II-tag antibody which recognised the uncleaved strep II-tag sequence remaining after the incomplete digestion of the protein **(Supplementary Fig. S1b**, lower panel**)**. The same sample of digested protein, however, gave a band of comparable intensity to that of undigested protein upon detection with $\alpha_2\delta$-1-specific antibody directed towards the N-terminus of the protein **(Supplementary Fig. S1b**, upper panel**)**. This confirms that the weaker bands detected using strep II-tag antibody after thrombin digestion are indeed due to the remaining uncleaved tag in protein molecules and not due to overall degradation of the protein.

Supplementary Fig. S2. Molecular weight determination of purified recombinant full-length TSPs and $\alpha_2\delta$-1$_S$ NTST by MALDI-TOF MS.

Full-length TSPs, i.e. TSP-2 **(a)**, TSP-4 **(b)** and COMP **(c)**, and $\alpha_2\delta$-1$_S$ NTST (+DTT, **e**) were pre-treated with 10 mM DTT in double-distilled H_2O overnight at 4 °C. Additional sample of $\alpha_2\delta$-1$_S$ NTST (-DTT, **d**) was investigated without DTT pre-treatment. MALDI-TOF MS was done with sinapinic acid as matrix. All spectra showed the signal of $[M+H]^+$ and $[M+2H]^{2+}$ of the target proteins. Furthermore, signals of $[M+3H]^{3+}$ **(c, d)** and $[2M+H]^+$ **(c)** were observed. BSA was used for external calibration of the spectra. Nevertheless, BSA signals were also detected in some spectra of target proteins, marked as * for signals of $[BSA+H]^+$ **(a, e)**.

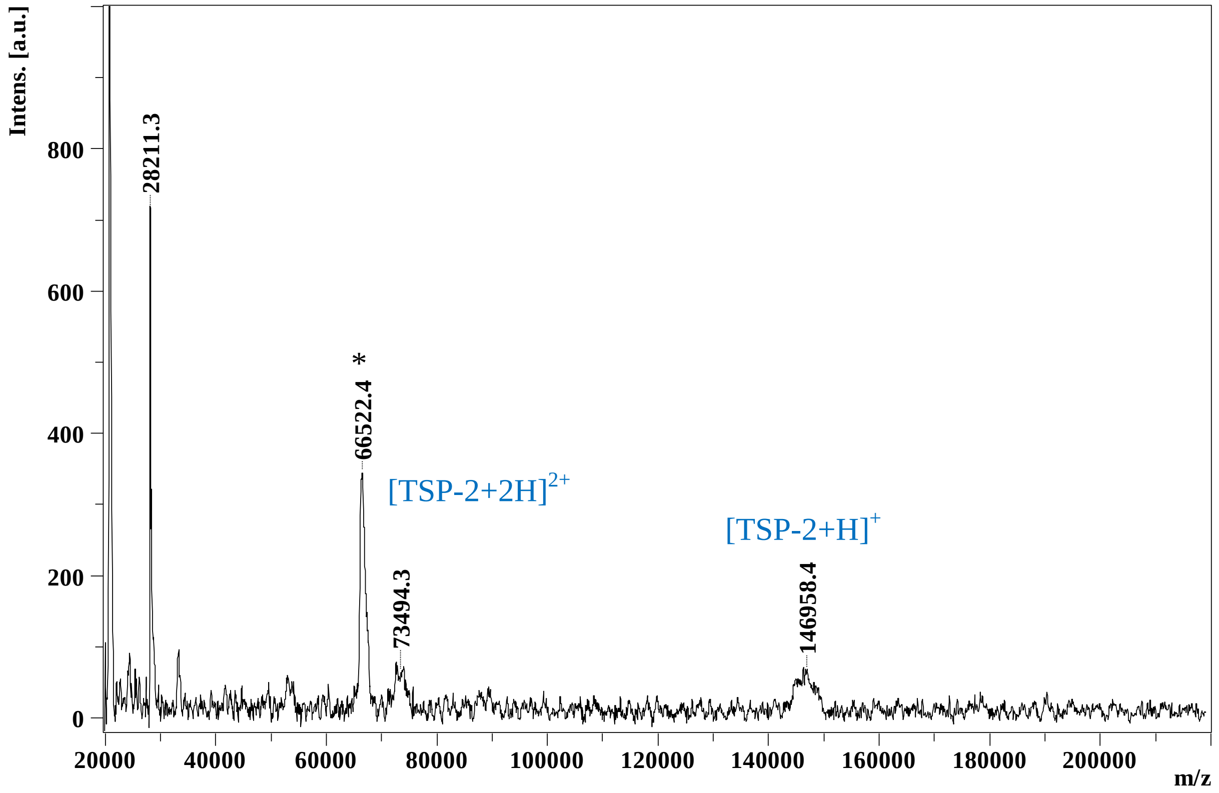
Intens. [a.u.]
800
600
400
200
0
28211.3
*
66522.4
[TSP-2+2H]2+
73494.3
[TSP-2+H]+
146958.4
20000
40000
60000
80000
100000
120000
140000
160000
180000
200000
m/z

Fig. S2a.

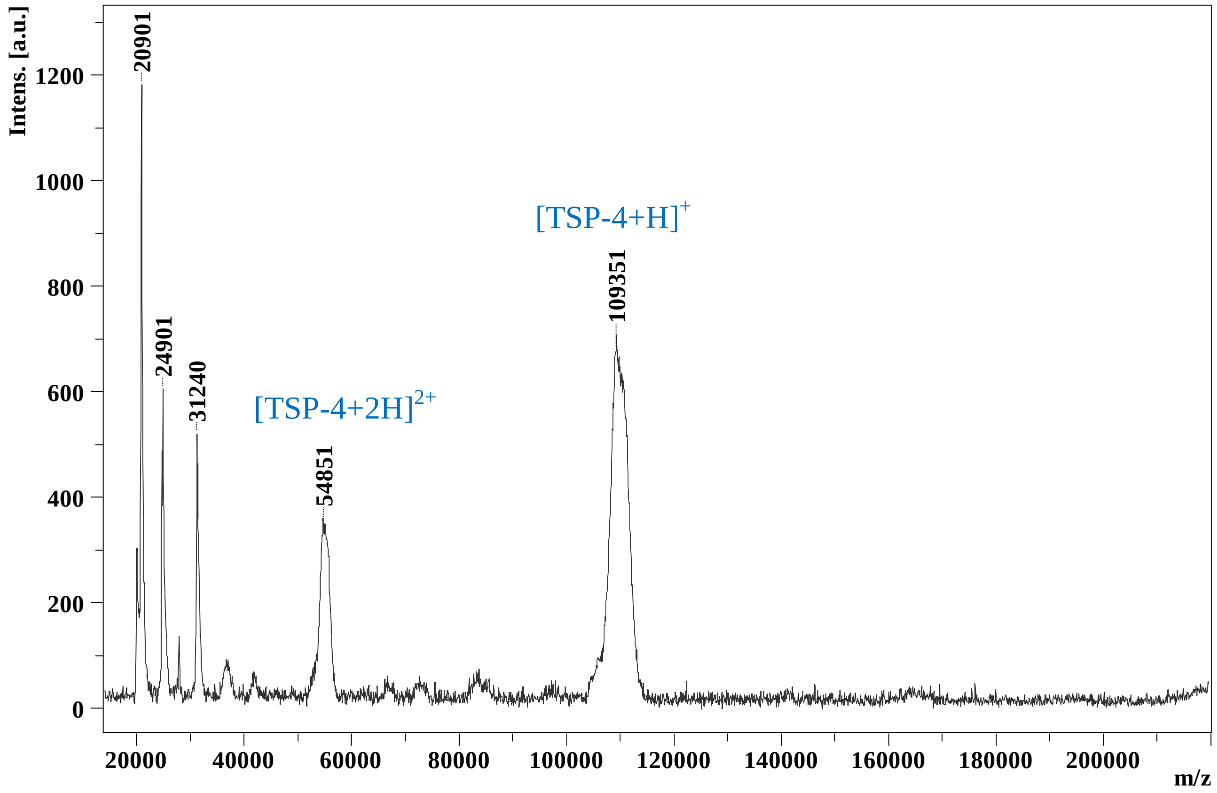
Intens. [a.u.]
1200
1000
800
600
400
200
0
20901
24901
31240
54851
109351
[TSP-4+2H]$^{2+}$
[TSP-4+H]$^{+}$
20000
40000
60000
80000
100000
120000
140000
160000
180000
200000
m/z

Fig. S2b.

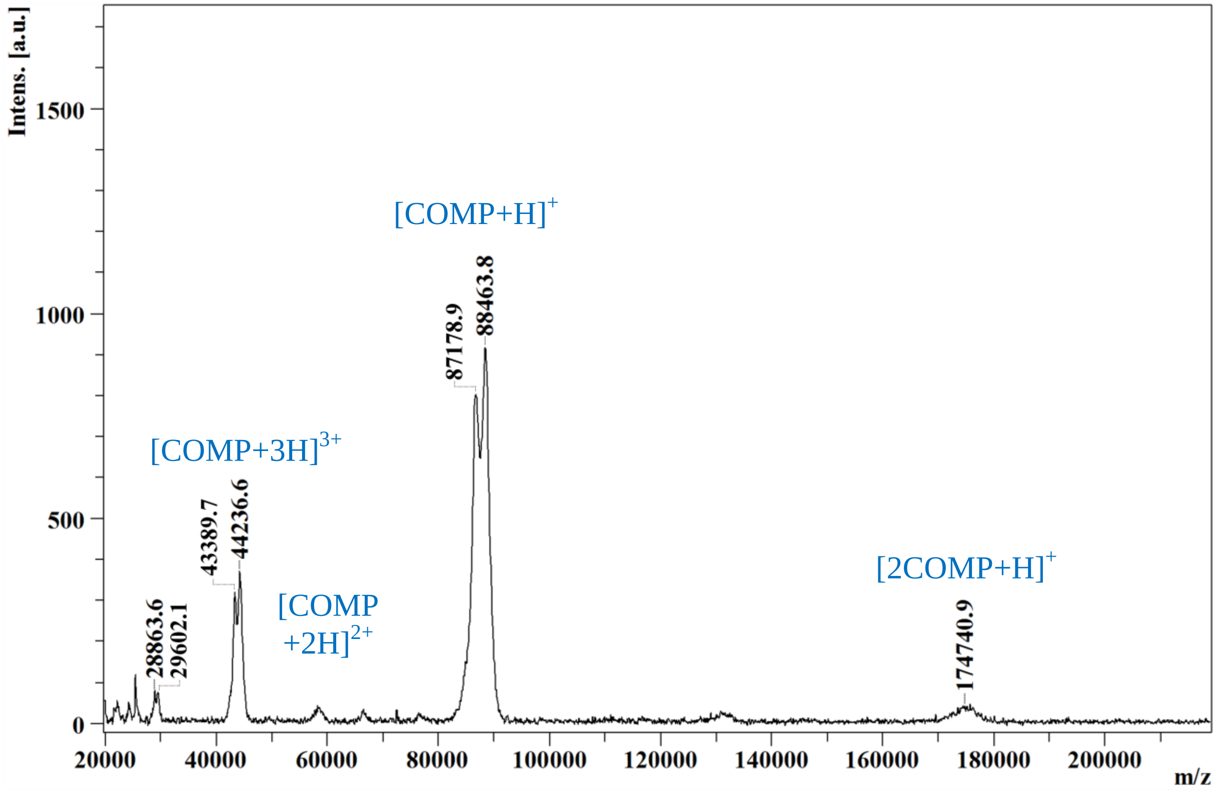

Intens. [a.u.]
1500
1000
500
0
[COMP+H]+
88463.8
87178.9
[COMP+3H]3+
43389.7
44236.6
[COMP
+2H]2+
28863.6
29602.1
[2COMP+H]+
174740.9
20000
40000
60000
80000
100000
120000
140000
160000
180000
200000
m/z

Fig. S2c.

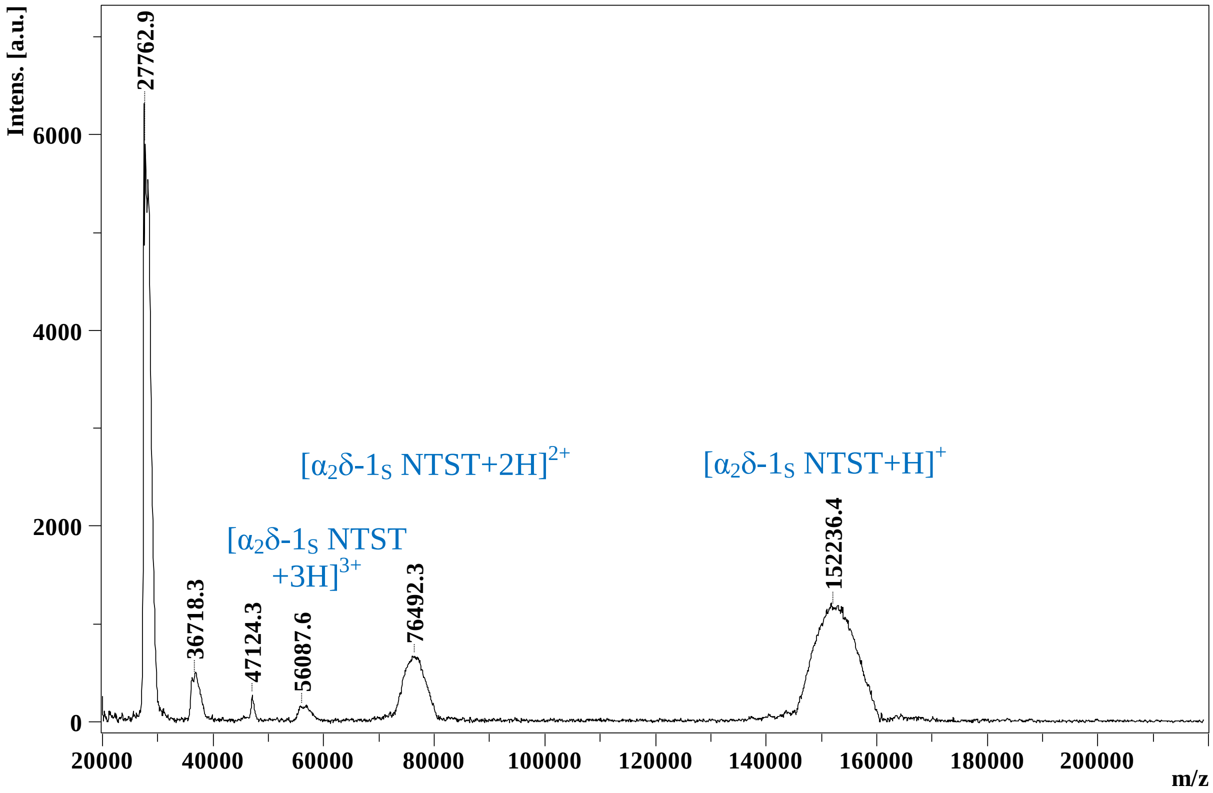

Intens. [a.u.]
6000
4000
2000
0
27762.9
36718.3
47124.3
56087.6
76492.3
152236.4
[α2δ-1S NTST+2H]2+
[α2δ-1S NTST+H]+
[α2δ-1S NTST
+3H]3+
20000
40000
60000
80000
100000
120000
140000
160000
180000
200000
m/z

Fig. S2d.

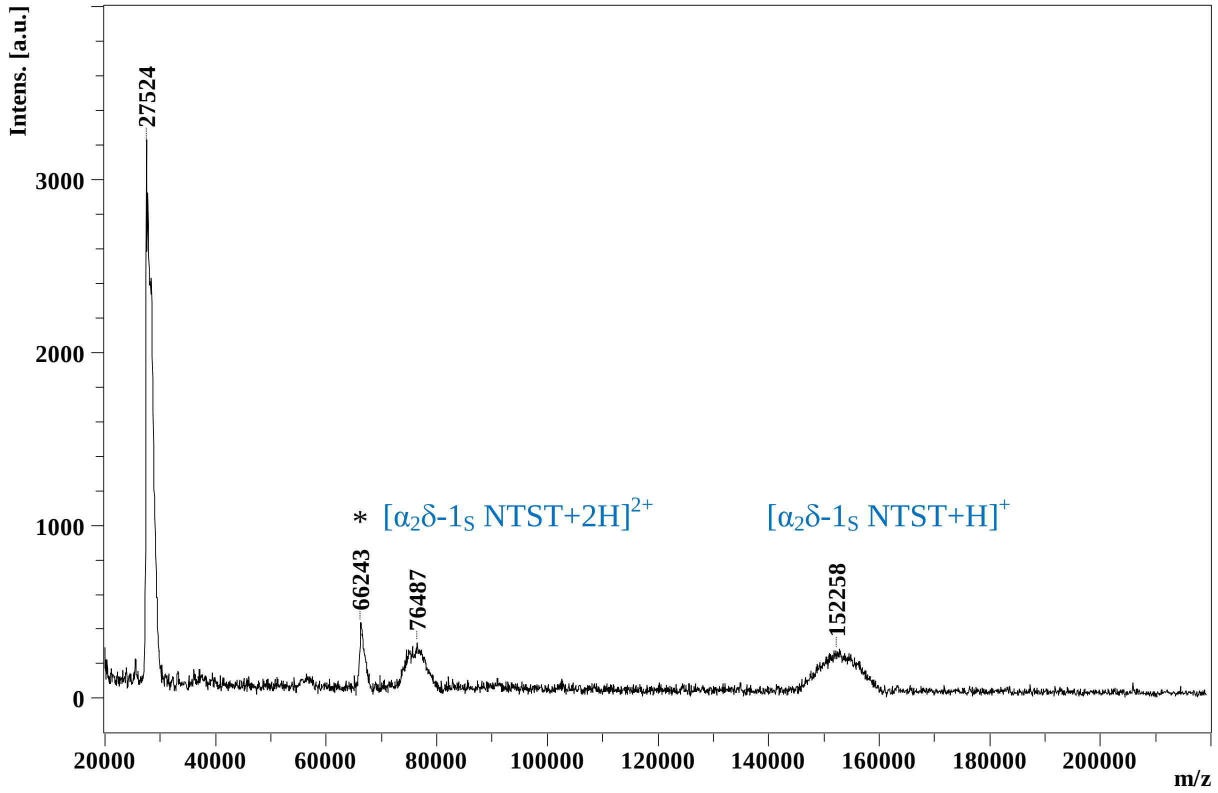
Intens. [a.u.]
3000
2000
1000
0
27524
66243
76487
152258
* [α2δ-1S NTST+2H]2+
[α2δ-1S NTST+H]+
20000
40000
60000
80000
100000
120000
140000
160000
180000
200000
m/z

Fig. S2e.

Supplementary Fig. S3. Surface plasmon resonance-based binding assay of GBP to captured $\alpha_2\delta$-1$_S$ NTST.

(a) A dilution series of GBP (31.25 nM, 62.5 nM, 125 nM, 250 nM, and 500 nM) in PBS pH 7.4 containing 0.05% Tween 20 was sequentially injected over the surface of a sensor chip prepared with $\alpha_2\delta$-1$_S$ NTST protein immobilised at 7395 (10 µg/ml) and 7928 RU (15 µg/ml) in the first and second experiments, respectively and at 8618 RU (15 µg/ml) for the third and fourth experiments. The corrected binding response at each GBP concentration after subtraction of non-specific binding response was assigned to RU at 4 sec before stopping the injection of the corresponding GBP concentration. **(b)** TOP: The corrected binding response, RU, was plotted versus the concentration of GBP, [GPB], and analysed by non-linear regression according to the equation $RU = RU_{max} * [GBP]/(K_D + [GBP])$, with RU_{max} and K_D being the maximum binding response and the dissociation constant of the two binding partners, respectively. Bottom: A Hanes-Woolf transformation of the data, i.e. plotting the ratio of [GBP] and RU versus [GBP], and a linear regression showed the linear interdependence of the transformed data and, thus, the equimolar binding of GBP to $\alpha_2\delta$-1$_S$ NTST.

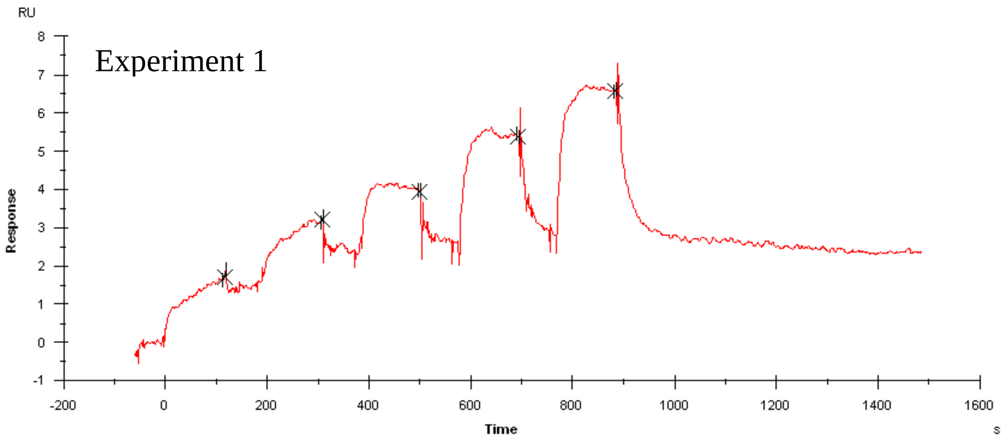

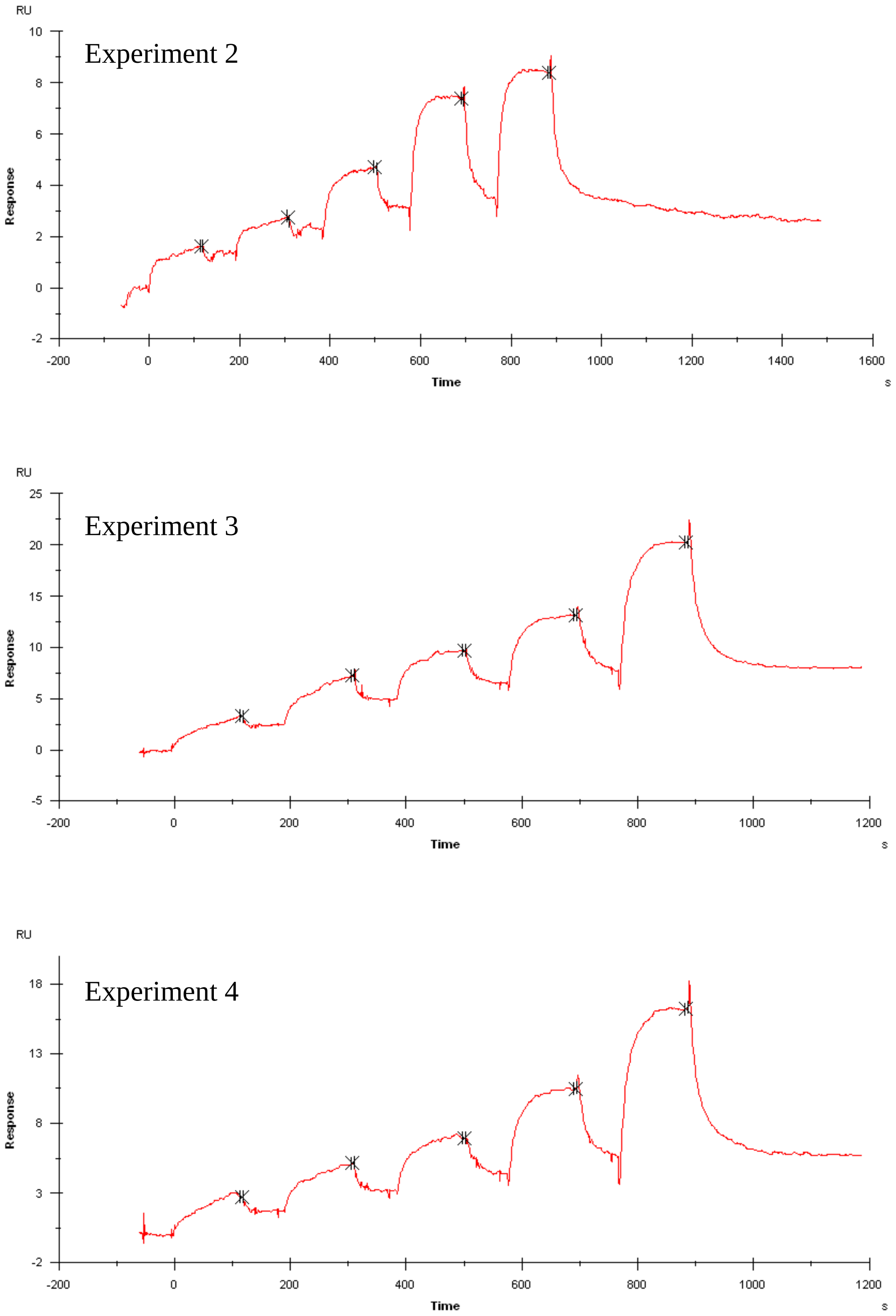
Experiment 2
RU
Response
Time
s
Experiment 3
RU
Response
Time
s
Experiment 4
RU
Response
Time
s

Fig. S3a

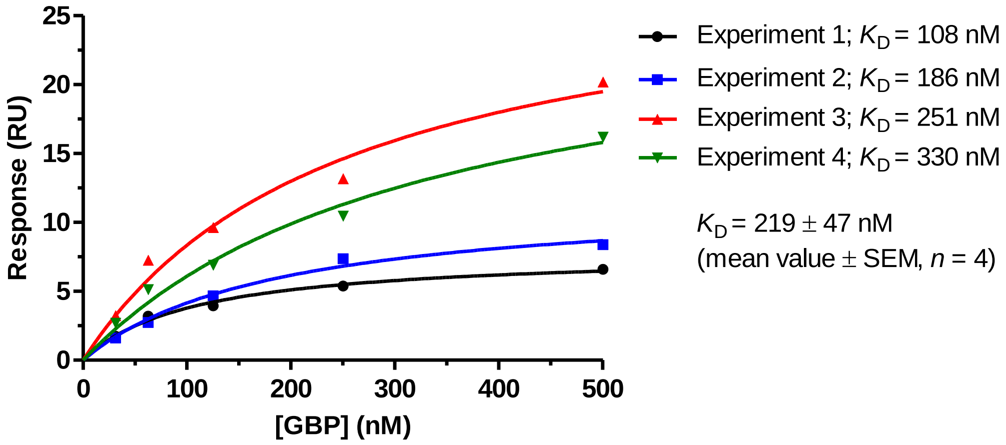

Response (RU)
25
20
15
10
5
0
0
100
200
300
400
500
[GBP] (nM)
Experiment 1; K_D = 108 nM
Experiment 2; K_D = 186 nM
Experiment 3; K_D = 251 nM
Experiment 4; K_D = 330 nM
K_D = 219 ± 47 nM
(mean value ± SEM, n = 4)

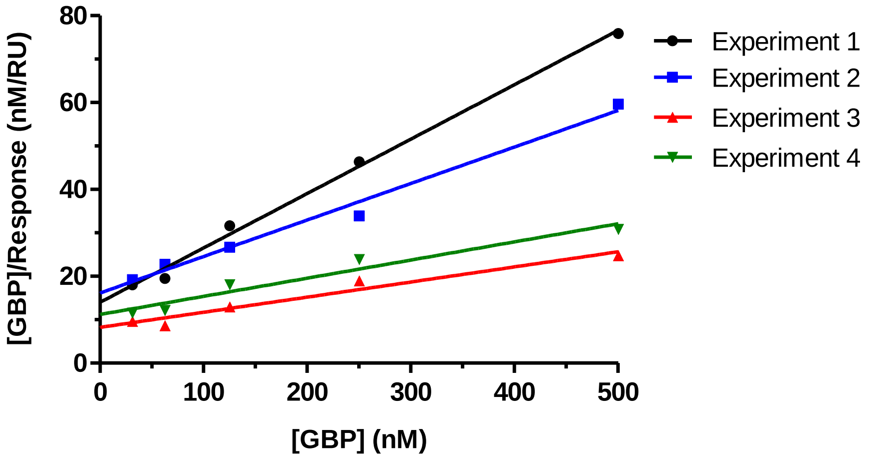

[GBP]/Response (nM/RU)
80
60
40
20
0
0
100
200
300
400
500
[GBP] (nM)
Experiment 1
Experiment 2
Experiment 3
Experiment 4

Fig. S3b.

Supplementary Fig. S4. Binding of recombinant COMP to matrilin-3 in an ELISA-style ligand binding assay.

50 µl of soluble recombinant matrilin-3 NTST (20 µg/ml) was coated onto 96-well plates and incubated with increasing concentrations of COMP (0.9-883 nM). Bound ligand was detected with specific COMP antiserum. Data of total and non-specific binding were used to calculate an apparent K_D value of 120 ± 15 nM (mean value ± SEM, n = 3), which is similar to the reported apparent K_D value of 56.3 nM obtained for the interaction of immobilized COMP (2 µg/ml) with soluble matrilin-3[7]. Data shown represent mean values ± SEM from three independent experiments, each performed in duplicate or triplicate.

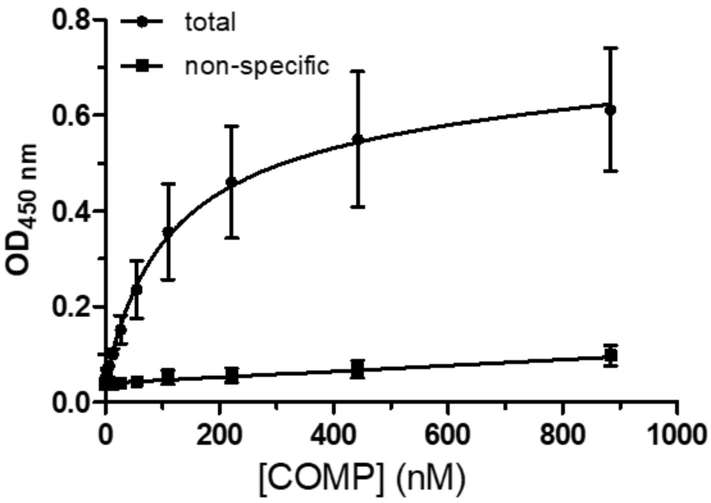

Fig. S4

Supplementary Fig. S5. Fluorescent A555-TSP-4 does not co-localise with over-expressed $\alpha_2\delta$-1 FL NTST in HEK293-EBNA cells.

Representative images of HEK293-EBNA cells transfected either with empty-(upper row) or $\alpha_2\delta$-1 FL NTST-expression vector (middle row, with the lower row representing a magnification of the image section within the red square in the left image of the middle row) after incubation with A555-TSP-4 (909 nM). Signals of $\alpha_2\delta$-1 FL NTST (left panel, grey and right panel, green), A555-TSP-4 (middle panel, grey and right panel, red) and DAPI (right panel, blue) are shown after membrane permeabilisation.

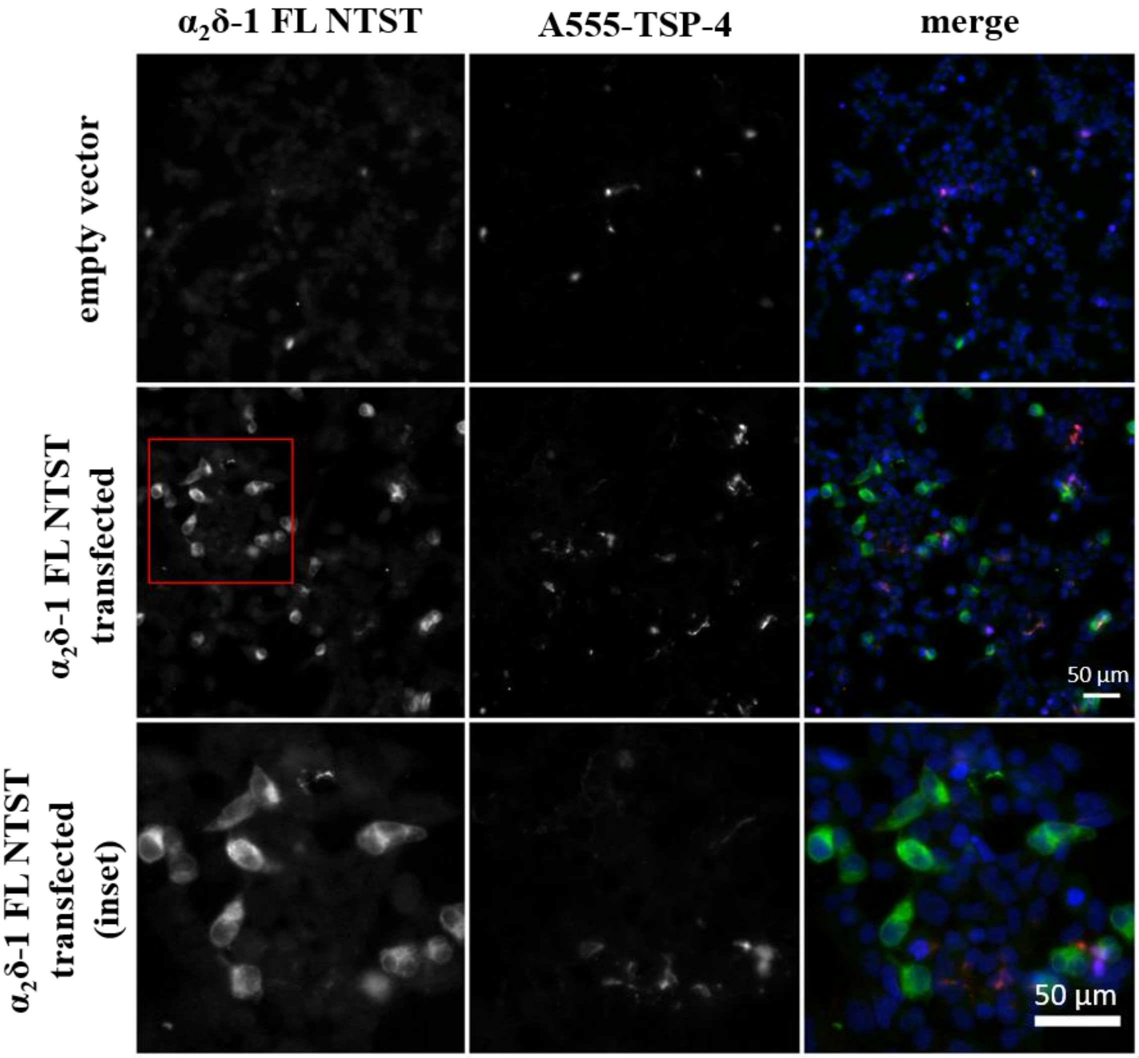
α2δ-1 FL NTST
A555-TSP-4
merge
empty vector
α2δ-1 FL NTST transfected
50 µm
α2δ-1 FL NTST transfected (inset)
50 µm

Fig. S5.

References

1 Sodersten, F. *et al.* Ultrastructural immunolocalization of cartilage oligomeric matrix protein, thrombospondin-4, and collagen fibril size in rodent achilles tendon in relation to exercise. *Connect Tissue Res* **48**, 254-262 (2007).

2 Kim, D. S. *et al.* Thrombospondin-4 contributes to spinal sensitization and neuropathic pain states. *J Neurosci* **32**, 8977-8987 (2012).

3 Dunkle, E. T., Zaucke, F. & Clegg, D. O. Thrombospondin-4 and matrix three-dimensionality in axon outgrowth and adhesion in the developing retina. *Exp Eye Res* **84**, 707-717 (2007).

4 Park, J. *et al.* Central Mechanisms Mediating Thrombospondin-4-induced Pain States. *J Biol Chem* **291**, 13335-13348 (2016).

5 Thur, J. *et al.* Mutations in cartilage oligomeric matrix protein causing pseudoachondroplasia and multiple epiphyseal dysplasia affect binding of calcium and collagen I, II, and IX. *J Biol Chem* **276**, 6083-6092 (2001).

6 Spitznagel, L., Nitsche, D. P., Paulsson, M., Maurer, P. & Zaucke, F. Characterization of a pseudoachondroplasia-associated mutation (His587-->Arg) in the C-terminal, collagen-binding domain of cartilage oligomeric matrix protein (COMP). *Biochem J* **377**, 479-487 (2004).

7 Otten, C. *et al.* A matrilin-3 mutation associated with osteoarthritis does not affect collagen affinity but promotes the formation of wider cartilage collagen fibrils. *Hum Mutat* **31**, 254-263 (2010).
